# Sequestration of Carbon Dioxide with Frustrated Lewis Pairs Based on N-Heterocycles with Silane/Germane Groups

**DOI:** 10.1021/acs.jpca.1c04787

**Published:** 2021-08-10

**Authors:** Maxime Ferrer, Ibon Alkorta, José Elguero, Josep M. Oliva-Enrich

**Affiliations:** † Instituto de Química Médica (CSIC), Juan de la Cierva, 3, 28006 Madrid, Spain; ‡ PhD Programme in Theoretical Chemistry and Computational Modelling, Doctoral School, Universidad Autónoma de Madrid, 28049 Madrid, Spain; § Instituto de Química-Física Rocasolano (CSIC), Serrano, 119, 28006 Madrid, Spain

## Abstract

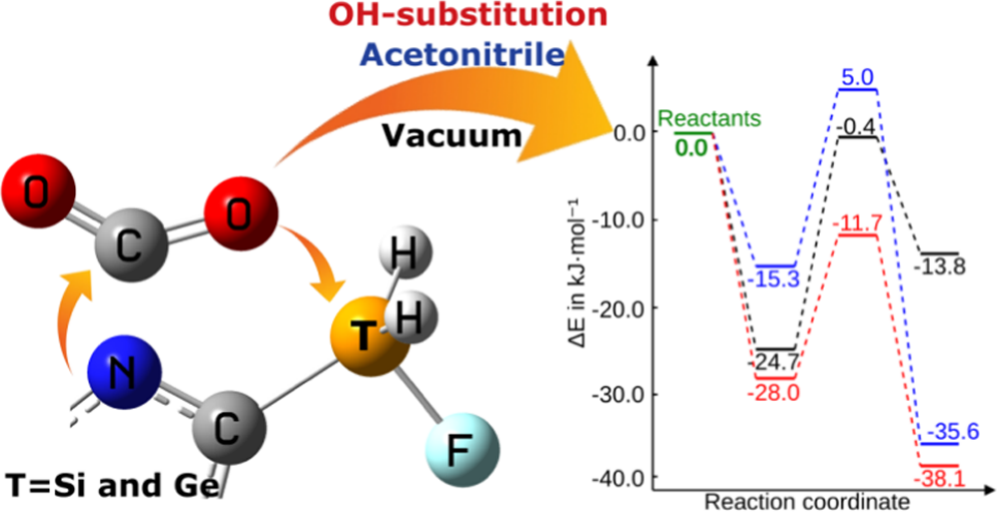

Frustrated Lewis pairs (FLPs) based on nitrogen heterocycles (pyridine, pyrazole, and imidazole) with a silane or germane group in the α-position of a nitrogen atom have been considered as potential molecules to sequestrate carbon dioxide. Three stationary points have been characterized in the reaction profile: a pre-reactive complex, an adduct minimum, and the transition state connecting them. The effect of external (solvent) or internal (hydroxyl group) electric fields in the reaction profile has been considered. In both cases, it is possible to improve the kinetics and thermodynamics of the complexation of CO_2_ by the FLP and favor the formation of adducts.

## 1 Introduction

Carbon dioxide, CO_2_, is a fascinating molecule. It is small, stable, and available in abundance; however, this apparently inoffensive compound has often its name written in capital letters next to the global warming phenomenon. Because of its greenhouse effect,^1−3^ the capture of carbon dioxide by accessible and cheap compounds is an active research area. There are a plethora of methods and patterns enabling the capture of CO_2_^4−7^ based on two major concepts: absorption and adsorption.^4^ In the adsorption process, the idea is to find compounds that, once they are organized into a surface, can create interactions with the carbon dioxide to capture it. The main goal here is to find a compound that is able to change the electronic distribution of CO_2_ to make this very stable compound a little more reactive. In previous works, it has been shown that carbon dioxide is able to form complexes with phosphines,^8−11^ sulfur dioxide,^12^ pyridine derivatives,^13−15^ imidazole,^16−18^ and other heterocycles.^19,20^ It has also been proved that CO_2_ can form adducts with carbenes^21−27^ and frustrated Lewis pairs (FLPs),^28−33^ some of them including silicon and germanium as Lewis acid centers.^34−36^

**Scheme 1:**
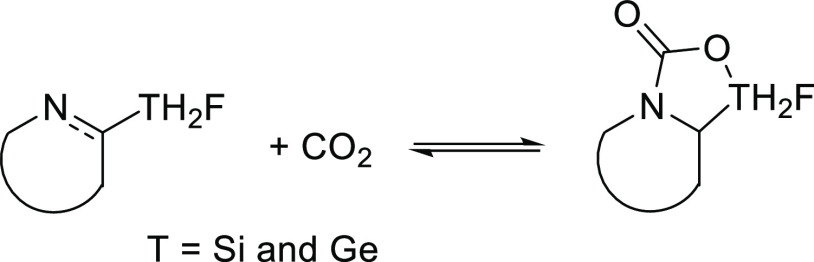
Simplified Model of the Reaction Studied between the N-Heterocyclic FLP and CO_2_

In this article, we explore the use of FLP based on N-heterocycles with a silane or germane group in the α-position of a nitrogen atom as potential molecules to form adducts with CO_2_ (Scheme 1). The nitrogen atom could act as a Lewis base (LB) and the silane or germane group as a Lewis acid (LA).^37−39^ The intramolecular disposition of the LA and LB adds rigidity to these systems, which should minimize the entropic effects. Derivatives of the studied molecules have been synthesized, and in some cases, their X-ray structure has been reported.^40−45^

Three stationary points have been characterized along the reaction coordinate between the FLPs and CO_2_: two minima, the pre-reactive complex and the adduct, and a TS connecting both minima. We explore the effect on the kinetics and thermodynamics of the reaction by means of an electric field generated by a solvent (external) and a hydroxyl group in the heterocyclic ring.

## 2 Computational Details

All of the structures presented in this work were optimized at the MP2 computational level^46^ with the jul-cc-pVTZ basis set.^47^ This basis set corresponds to the aug-cc-pVTZ^48^ for all atoms except hydrogen where cc-pVTZ is used. The energy minima and TS structures (zero and one imaginary frequency, respectively) were confirmed by frequency computations. The Gaussian-16 scientific software^49^ was used in these calculations, and the coordinates of the stationary points are gathered in Table S1 of the Supporting Information (SI).

The solvent effect was simulated using the PCM model^50^ with the dielectric constant for acetonitrile (ϵ = 35.69). This solvent was selected based on previous studies that show a small complexation with the FLP before reacting with CO_2_, which could prevent the adduct formation between FLP and CO_2_.^51^

The electron density of the systems was analyzed within the quantum theory of atoms in molecules (QTAIM)^52,53^ and AIMAll software.^54^ Based on this method, the electron density critical points are located and, using the signature of the second derivative (Laplacian), these critical points can be classified as nuclear attractors, bond, ring, and cage critical points. The characteristics of the bond critical points (BCP) provide important information about the contact between the two atoms involved.

We used the NBO method,^55^ with the NBO-7 version^56^ of the program connected with the Gaussian-16 program, to evaluate the stabilization due to the charge transfer between occupied and empty orbitals specially in intermolecular interactions. These calculations were carried out with the M06-2X functional^57^ with the geometries obtained at the MP2 level to account for electronic correlation.

## 3 Results and Discussion

**Figure 1:**
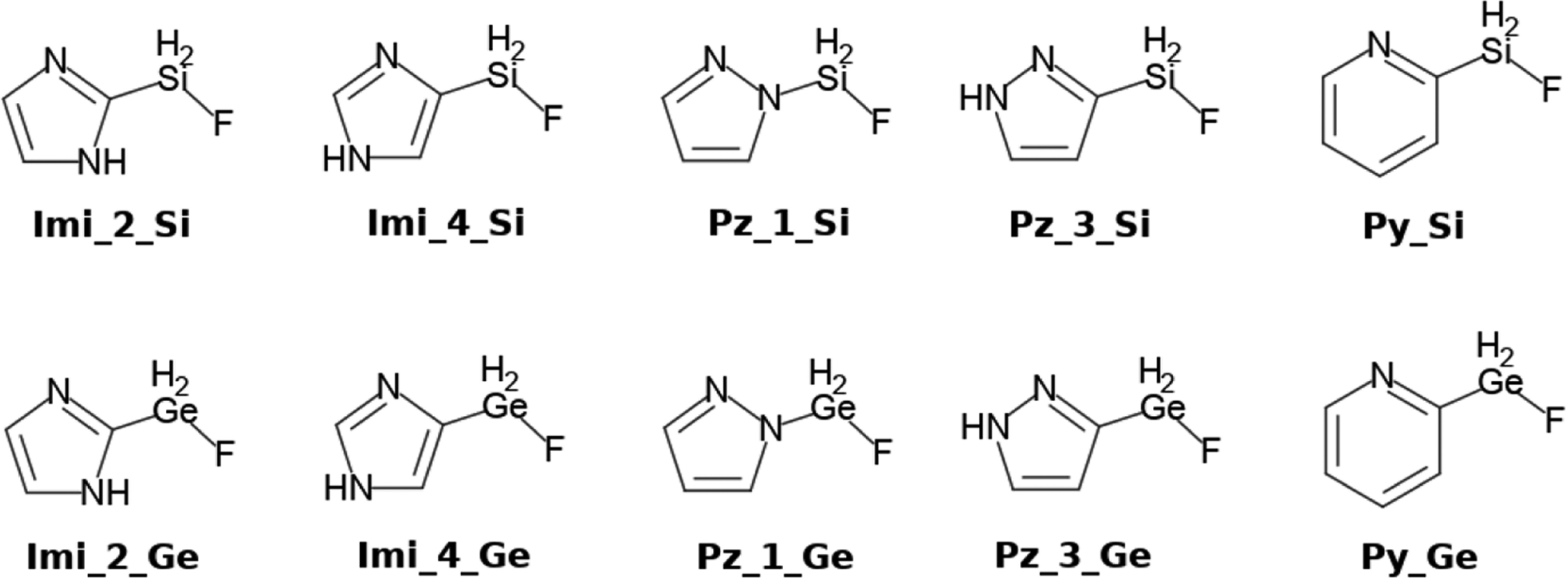
Ten FLP molecules considered in the present article with the indication of the nomenclature used.

We study the formation of adducts between CO_2_ and 10 FLP molecules based on N-heterocycles with silane and germane groups displayed in Figure 1. This section is divided into four parts. The first part analyzes the electronic properties of the isolated FLP and CO_2_ molecules. In the second part, the three stationary points of the reaction of the FLP + CO_2_ in a vacuum are considered. In the third section, we discuss the solvent effect, and the last section is focused on the effect of including a hydroxyl group near the GeH_2_F moiety.

### 3.1 Electronic Properties of the Isolated FLP

**Figure 2:**
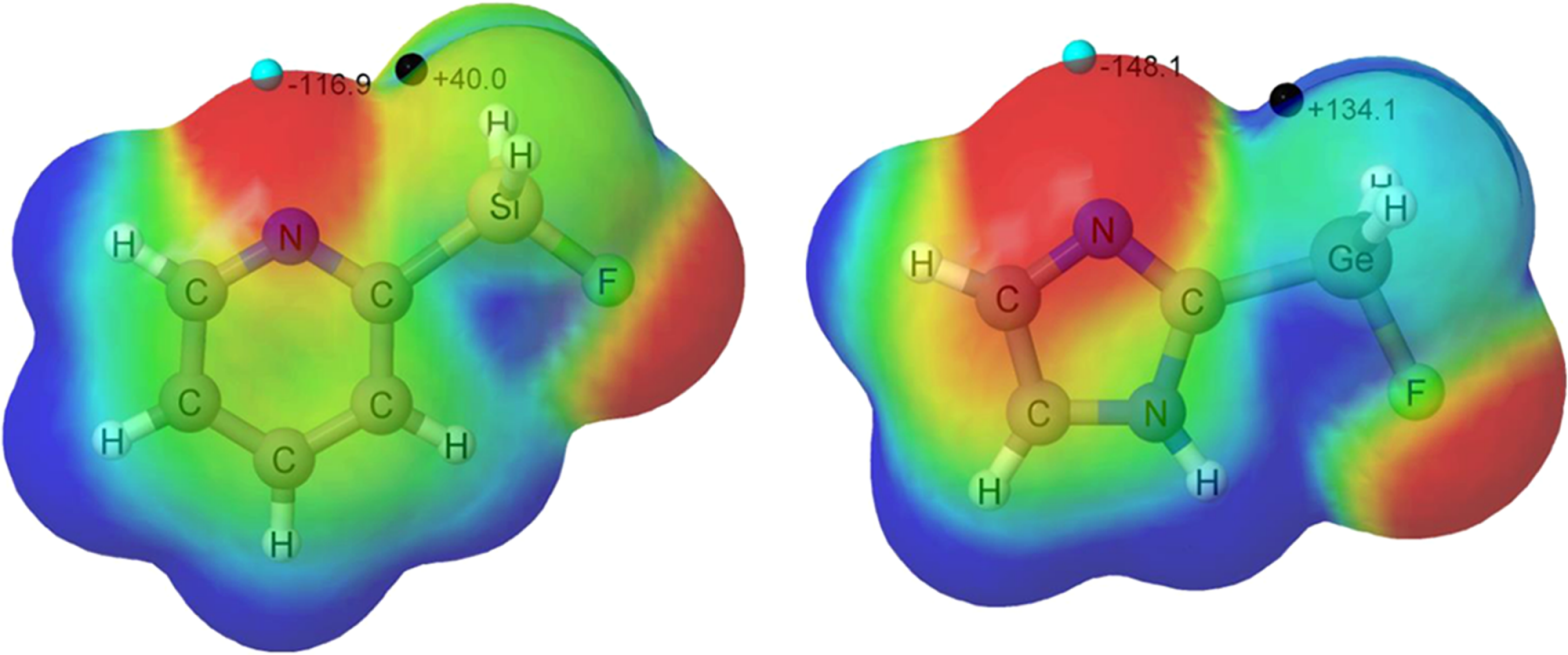
Molecular electrostatic potential on the 0.001 au electron density isosurface of Py_Si (left) and Imi_2_Ge (right). The location of the minimum associated to the N lone pair and maximum corresponding to the Si/Ge–F σ hole are indicated by light-blue and black spheres, respectively. The value of the maximum and minimum is given in kJ mol^–1^.

The isolated FLP considered in this work present a molecular electrostatic potential (MEP) suitable for simultaneous interactions of one of the nitrogen atoms as a Lewis base (negative values of MEP) with the carbon atom of CO_2_ and the silane/germane group as a Lewis acid (positive values of MEP, σ-hole^58,59^) with one of the oxygen atoms of CO_2_. Two examples are depicted in Figure 2, and the extreme values associated with the Lewis acid and Lewis base centers of the FLP are listed in Table 1.

**Table 1:** Minima and Maxima of the MEP (kJ mol^–1^) on the 0.001 au Electron Density Isosurface Associated with the N and Silane/Germane Groups, Respectively

FLP	*V*_s,min_ (N)	*V*_s,max_ (Si–F)	FLP	*V*_s,min_ (N)	*V*_s,max_ (Ge–F)
Imi_2_Si	–153.3	99.6	Imi_2_Ge	–148.1	134.1
Imi_4_Si	–150.6	63.8	Imi_4_Ge	–143.9	96.7
Pz_1_Si	–119.0	79.2	Pz_1_Ge	–116.6	119.0
Pz_3_Si	–117.4	81.7	Pz_3_Ge	–110.2	109.0
Py_Si	–116.9	40.0	Py_Ge	–108.3	79.6

The negative values of the extreme MEPs associated with nitrogen in the FLPs range between −108 and −153 kJ mol^–1^, being larger in imidazole than in pyrazole and pyridine derivatives; the two latter show similar values. The σ-hole associated to the Si–F/Ge–F bond presents positive values of the extreme MEPs between 40 and 134 kJ mol^–1^. The largest and smallest values in the two series (Si and Ge derivatives) correspond to the Imi_2 and Py compounds, respectively. The replacement of Si by Ge in FLPs increases the extreme MEP values of the σ-hole, by 35 kJ mol^–1^ on average, while the absolute value of the minimum associated with the lone pair in nitrogen decreases only by 6 kJ mol^–1^. Thus, we expect that the Ge derivatives should form stronger complexes with CO_2_ as compared to the complexes of the corresponding Si derivatives.

### 3.2 FLP + CO_2_ Reaction in Gas Phase

**Figure 3:**
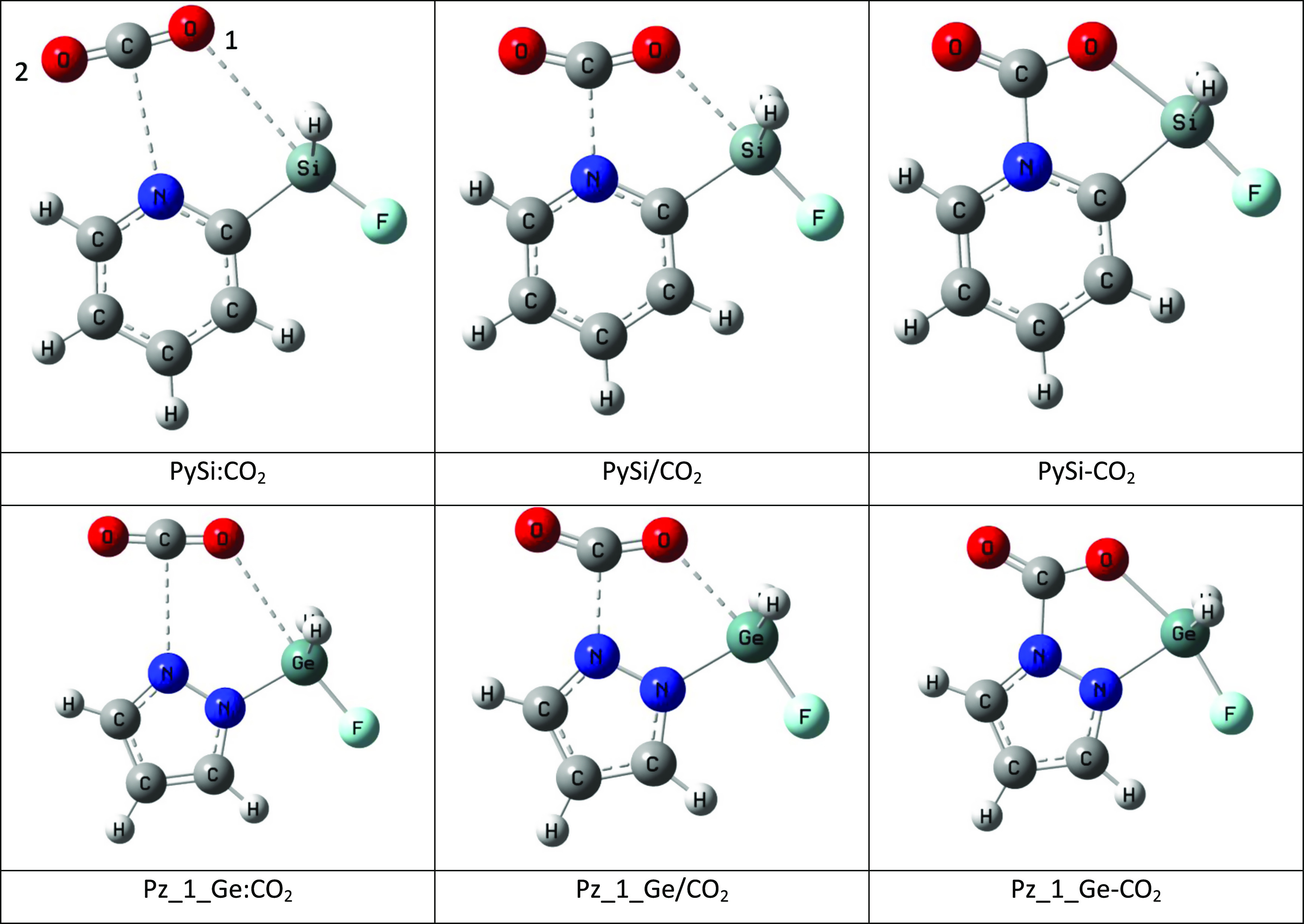
Stationary points of the PySi + CO_2_ and Pz_1_Ge + CO_2_ systems. The numbering used to identify the two oxygens of CO_2_ has been indicated.

Three stationary points were characterized between the FLP and CO_2_. Initially, an energy minimum complex was obtained between the two systems that can evolve through a TS to an adduct (see Figure 3 for two examples). Only in the case of the Pz_3_Ge + CO_2_ system, the adduct was not located and all of the attempts to locate it evolved spontaneously toward the complex. The following nomenclature will be used in this article to differentiate the three stationary points: FLP:CO_2_ for the complex, FLP/CO_2_ for the TS, and FLP–CO_2_ for the adduct, that is :, /, and −.

**Figure 4:**
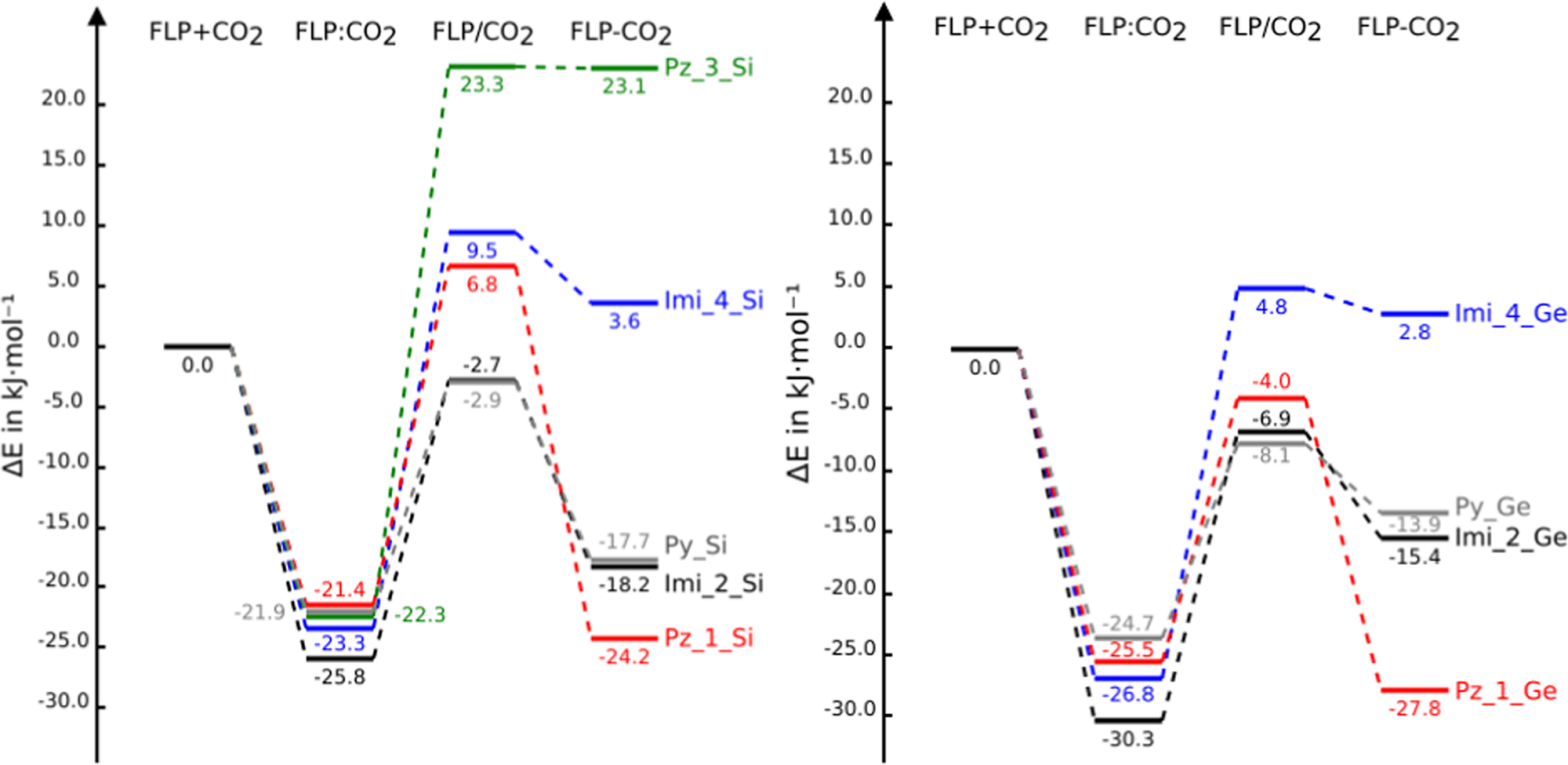
PES of the FLP + CO_2_ reaction profiles. Energy profiles for silicon and germanium derivatives are shown on the left and right, respectively.

The N···C and O···Si/Ge interatomic distances in the three stationary points are gathered in Table 2 and the relative energies with respect to the isolated monomers along the reaction coordinate are represented in Figure 4 and listed in Table S2 of the SI. The profile of the free energy evolution (Figure S1) is similar to Figure 4 but with more positive values due to the entropic effects. A graphical representation of the evolution of four interatomic distances [N–C, O–Si/Ge, C–O(1), and C–O(2)] along the reaction coordinate is included in the SI (Figure S2).

**Table 2:** Selected Interatomic Distances in the Three Stationary Points Found in the Gas Phase (Å)

	FLP:CO_2_		FLP/CO_2_		FLP–CO_2_	
FLP	N···C	O(1)···Si/Ge	N···C	O(1)···Si/Ge	N–C	O(1)–Si/Ge
Imi_2_Si	2.695	2.908	1.892	2.363	1.522	2.035
Imi_4_Si	2.725	3.093	1.802	2.434	1.545	2.141
Pz_1_Si	2.772	3.256	1.954	2.305	1.495	1.927
Pz_3_Si	2.727	3.219	1.670	2.517	1.593	2.397
Py_Si	2.763	3.286	1.984	2.450	1.577	2.041
Imi_2_Ge	2.677	2.804	1.862	2.440	1.547	2.201
Imi_4_Ge	2.704	2.930	1.774	2.498	1.584	2.334
Pz_1_Ge	2.721	3.008	1.945	2.385	1.506	2.057
Pz_3_Ge	2.719	3.013				
Py_Ge	2.702	3.081	1.948	2.472	1.624	2.210

#### 3.2.1 FLP:CO_2_ Complexes

The FLP:CO_2_ complexes present two tetrel bond interactions,^60−63^ with both molecules acting simultaneously as a tetrel donor and an acceptor, which could favor a cooperative effect. The two intermolecular distances that characterize the interactions range between 2.77 and 2.68 Å for N···C and between 2.80 and 3.29 Å for the O···Si/Ge ones. The shortest distances in both series are found in the Imi_2 complexes. Both tetrel bond distances in the Ge series are shorter than in the Si one, in agreement with the MESP results of the isolated FLP molecules discussed previously.

The binding energies of the FLP:CO_2_ complexes range between −30 and −21 kJ mol^–1^, which is similar to other complexes involving CO_2_.^10,19,24,25^ In agreement with the MESP values and the intermolecular distances, the strongest complexes in both series correspond to the Imi_2 complexes. The complexes in the Ge series are on average 3.5 kJ mol^–1^ stronger than the corresponding ones in the Si series. These results are in agreement with previous reports that have shown that Ge is a better tetrel bond donor than Si.^62^

#### 3.2.2 FLP–CO_2_ Adducts

The second minimum found in the reaction coordinate corresponds to the FLP–CO_2_ adducts. As indicated previously, in the case of Pz_3_Ge–CO_2_, all attempts to locate an adduct spontaneously evolve toward the complex previously discussed. In these minima, the N–C distances range between 1.50 and 1.62 Å and the O(1)–Si/Ge distances between 1.93 and 2.40 Å. The Si/Ge atoms are penta-coordinated with a bipyramidal arrangement. The shortest distances are found in the Pz_1 adducts in both series while the longest, in the Si series, correspond to the Pz_3_Si–CO_2_ adduct, not present in the Ge series as mentioned above. As opposed to the trends observed in the complexes, shorter distances are found in the adducts of Si compounds as compared to the Ge series for both N–C and O–Si/Ge parameters.

The relative energies of the adducts range between +23 and −28 kJ mol^–1^. They can be divided into three groups:

- Less-stable adducts than the corresponding isolated reactants: Pz_3_Si, Imi_4_Si, and Imi_4_Ge.
- More-stable adducts than isolated reactants but less-stable adducts than complexes: Imi_2_Si, Imi_2_Ge, Py_Si, and Py_Ge.
- More-stable adducts than reactants and complexes: Pz_1_Si and Pz_1_Ge.

#### 3.2.3 FLP/CO_2_ TS

In the TS structures there are intermediate N–C (between 1.77 and 1.98 Å) and O(1)–Si/Ge distances (between 2.30 and 2.52 Å) as compared to those found in the corresponding two energy minima. The N–C distances are shorter in the TS of the Ge series as compared to those corresponding in the Si series while the opposite happens with the O–Si/Ge distances, shorter in the Si series than in the Ge one.

With respect to the energies of the TS, they can be divided into two groups:

- Positive relative energies (less stable than the isolated reactants) are found in four cases: imi_4_Si, Pz_1_Si and Pz_3_Si, and imi_4_Ge.
- Negative relative energies (more stable than the isolated reactants) are found in five cases: Imi_2_Si/Ge, Imi_2_Ge, Pz_1_Ge, Py_Si, and Py_Ge.

The activation barriers (energy difference between the adduct and the TS) range between 17 and 47 kJ mol^–1^.

#### 3.2.4 Overall Analysis in Gas Phase

The geometrical and energetic values of the stationary points along the reaction coordinate have been used to evaluate the two parameters γ and β, defined in eqs 1 and 2, respectively, proposed by Cioslowski^64^ to approach the Hammond postulate.^65^ The γ parameter defines the exothermicity using the energies of the complex, adduct, and TS. The β parameter evaluates the geometrical proximity of the reactants to the transition state using three Euclidean distances (C–O(1), C–N, and O–Si/Ge)^66^
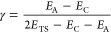
1
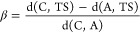
2where A and C indicate adducts and complexes, respectively. The values obtained for the two parameters have been gathered in Table S3.

The two parameters can vary between −1.0 and +1.0. Exothermic reactions correspond to negative values of γ (here only those involving Pz_1_Si and Pz_1_Ge, −0.05 in both cases), while endothermic reactions show positive values of γ (all other reactions with values up to 0.81). In the present case, only two reactions are exothermic (those involving Pz_1_Si and Pz_1_Ge) with negative but small values of γ (Table S3). The value of β will be close to 1 when the geometries of adducts and TS are very similar; otherwise, the values of β are close to −1 when the geometries of the TS and complexes are similar. In the present case, they range between 0.29 and 0.81, which indicates that all of the TS geometries are more similar to the adducts than the complexes as indicated by the positive values of β. In this set of parameters, the largest values of γ are associated to those of β, showing a second order polynomial relationship between the two parameters (*R*^2^ = 0.97).

As regards to the electron density properties, the values of the electron density properties at the bond critical points of the two tetrel contacts in the stationary points along the reaction coordinate (Table S4) were characterized using the properties at the N–C and Si/Ge–O BCPs (Table S5). Thus, three parameters at the BCP are used for the classification of the contacts as proposed by Mata et al.:^67^

- ∇^2^ρ(*r*)_BCP_ > 0; *H*_BCP_ > 0; |*V*_BCP_| = *G*_BCP_ < 1: closed-shell interaction (CSI).
- ∇^2^ρ(*r*)_BCP_ > 0; *H*_BCP_ < 0; 1 < |*V*_BCP_| = *G*_BCP_ < 2: closed-shell interaction with a significant covalent character (CSI-COV).
- ∇^2^ρ(*r*)_BCP_ < 0; *H*_BCP_ < 0; |*V*_BCP_| = *G*_BCP_ > 2: covalent interactions (COV).

Based on the above parameters, all of the BCPs in the complexes are CSI, changing to CSI-COV in the TS except for two N–C contacts: Pz_3_Si and Imi_4_Ge, both corresponding to COV. Finally in the adducts, all the N–C bonds are characterized as COV while the Si/Ge–O are CSI-COV. These results are also corroborated by the values of the electron density at the BCPs along the reaction coordinate (Table S4). They are small in the complexes (around 0.015 and 0.010 au for the N–C and Si/Ge–O BCPs, respectively), increase in the TSs (between 0.16 and 0.09 au for the N–C BCPs and between 0.03 and 0.02 au for the Si/Ge–O BCPs), showing the largest values in the adducts (between 0.24 and 0.19 au for the N–C BCPs and between 0.07 and 0.03 au for the Si/Ge–O BCPs). Excellent exponential correlations (*R*^2^ > 0.99) are found between the values at the BCP and the interatomic distances for the N–C, Si–O, and Ge–O contacts in the whole range of distances (Figure S3) in agreement with previous reports.^68−70^

**Figure 5:**
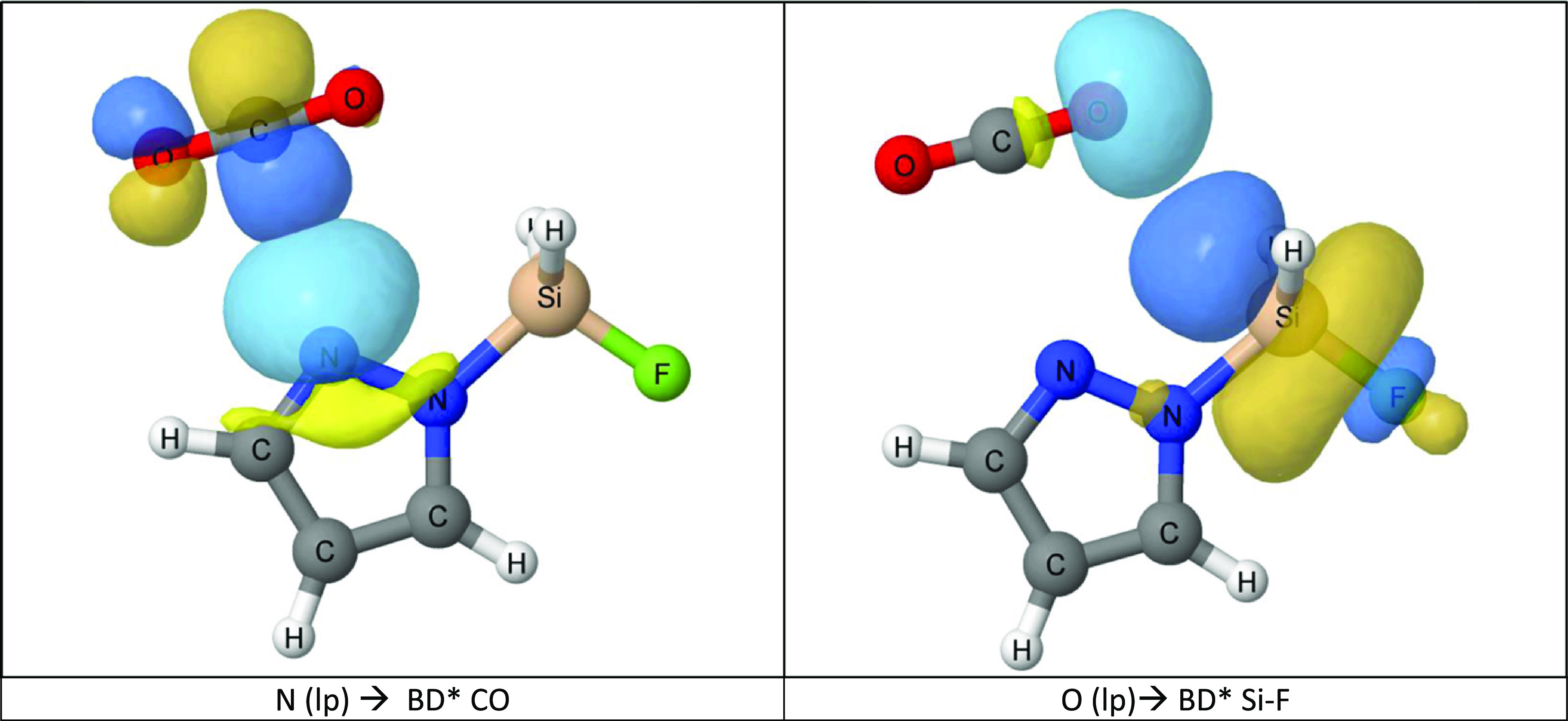
Two most important orbital charge-transfer interactions of the two molecules along the reaction coordinate are shown for the Pz_1_Si:CO_2_ complex.

The NBO analysis shows that two interactions between occupied and empty orbitals are the most important to explain the attractive forces between the two molecules along the reaction coordinate, the N(lp) → BD* CO and O(lp) → BD* Si/Ge–F. They have been represented in Figure 5 for the Pz_1_Si:CO_2_ complex. In agreement with the QTAIM results, the NBO charge-transfer stabilization energies (Table S6) increases when going from the complexes to the adducts through the TS, with C–N contacts considered as bonded in all adducts and the Pz_3_Si and Imi_4_Ge TS structures.

### 3.3 Solvent Effects

The study of the reaction in gas phase shows very different dipole moments in complexes (between 0.27 and 4.31 D) and adducts (between 4.12 and 8.86 D). Thus, a priori, the presence of solvents could stabilize in a larger degree the adducts as compared to the complexes, thus changing the energy profiles in the reaction. Consequently, the effect of acetonitrile was considered for all cases using the PCM model.

**Figure 6:**
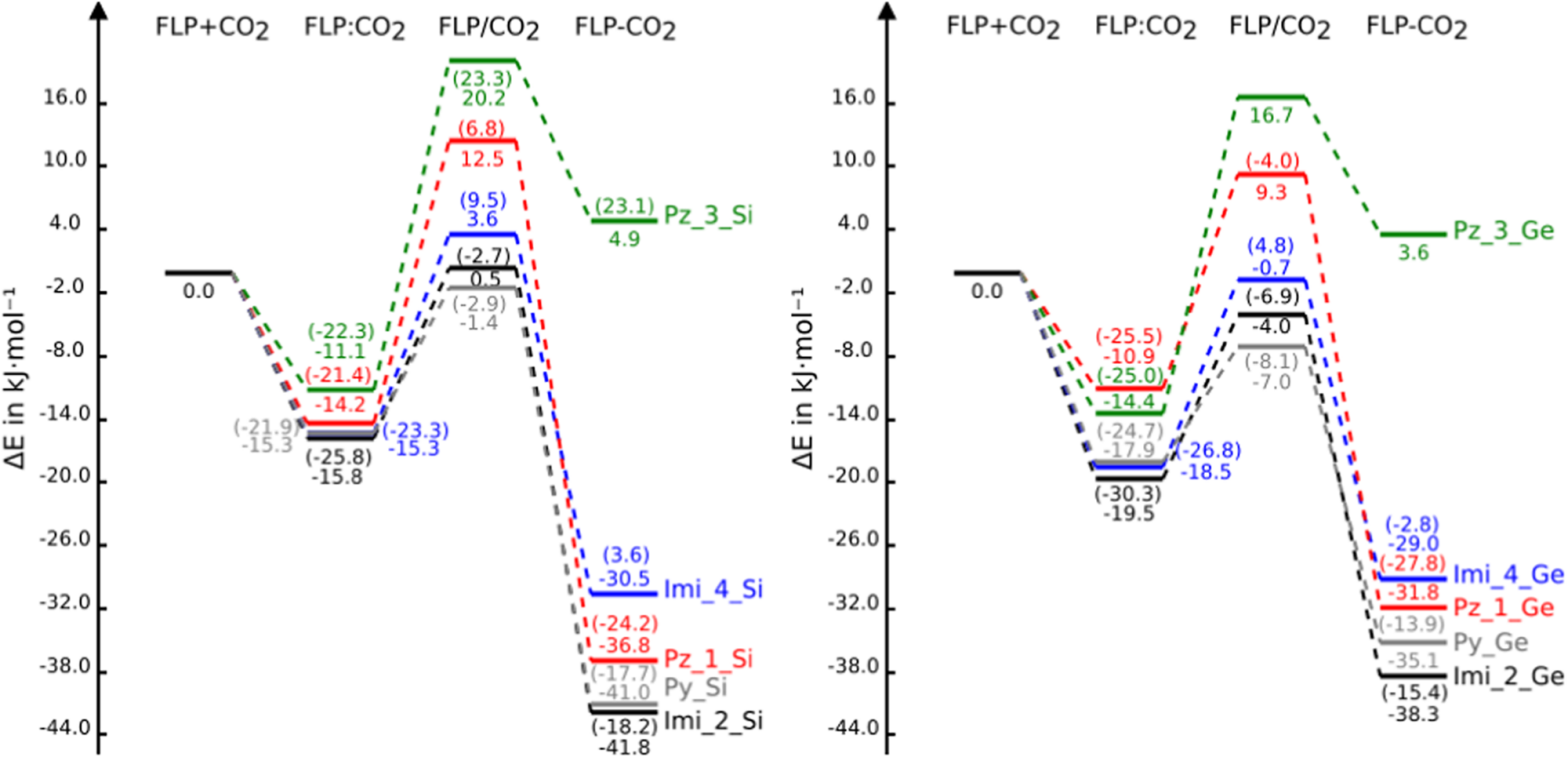
PES of the FLP + CO_2_ reaction profiles including the effect of the solvent [PCM (acetonitrile)]. The values in parenthesis are those corresponding to the reaction in gas phase. Silicon and germanium derivatives are shown on the left and right panels, respectively.

The solvent effect destabilizes the complexes by 9 kJ mol^–1^ on average when compared to the analogous results in gas phase (Figure 6 and Table S7). In contrast, the adducts in the PCM model (acetonitrile) are more stable by 23 kJ mol^–1^ on average than without solvent. In addition, the inclusion of the solvent model allows to locate the adduct of Pz_3Ge, which could not be located in the gas phase.

Another interesting effect of the solvent is that now, in the PCM model (acetonitrile), all of the adducts are more stable than the complexes and the isolated monomers, except for Pz_3_Si and Pz_3_Ge, where the adducts present relative energy of +4.9 and +3.6 kJ mol^–1^, respectively.

The overall effect of the solvent on the energy and geometry of the stationary points are reflected in the calculated γ and β parameters that are smaller (more negative in the case of γ) in the PCM (acetonitrile) to the ones in the gas phase (Table S8). In the PCM model, the values of the γ parameter are negative since all of the reactions are exothermic, and the β ones are positive but small, an indication that the TS geometries are intermediate between the two minima. The only exception is the reaction involving Pz_3_Ge that show positive values of γ (+0.41) and a value of β of 0.64.

The ρ_BCP_ values (Table S9) obtained for the intermolecular interactions using the PCM (acetonitrile) model are slightly smaller than those found in the complexes and TS in gas phase, in agreement with the longer interatomic distances found when the system is solvated than in vacuo. In contrast, the ρ_BCP_ values in the adducts are slightly larger since in this case, the bond distances are sorter when solvated than in vacuo.

**Figure 7:**
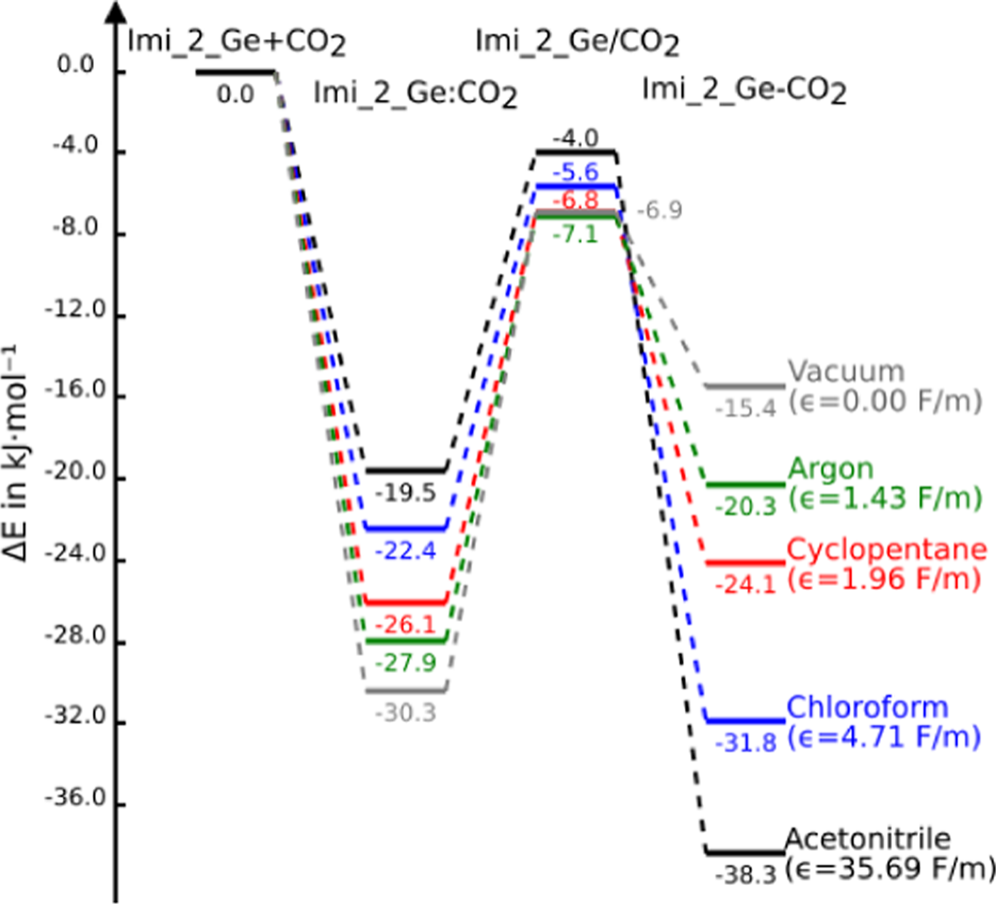
Relative energy (kJ mol^–1^) of the stationary points of the Imi_2_Ge + CO_2_ reaction as a function of some selected solvents with the PCM model.

To get more insight into the effect of the solvent in the energy profile, the effect of eight solvents with a large variety of dielectric constants was considered in the Imi_2_Ge + CO_2_ reaction. We considered the following solvents: argon (ϵ = 1.43 F/m), pentane (ϵ = 1.84 F/m), cyclopentane (ϵ = 1.96 F/m), dibutylether (ϵ = 3.0 F/m), chloroform (ϵ = 4.71 F/m), octanol (ϵ = 9.86 F/m), acetone (ϵ = 20.49 F/m), and acetonitrile (ϵ = 35.69 F/m). A clear relationship is obtained between the inverse of the dielectric constant of the solvent and the reaction energy (Figure S4). The larger the dielectric constant, the more exothermic the reaction is (in Figure 7, we display the energy profile for the five selected solvents). In addition, a reduction of the activation energy is observed following the postulate of Laidler and Landskrener that relates this parameter with (1 – 1/ϵ).^71^ In fact, a linear correlation between *E*_a_ and (1 – 1/ϵ) is obtained with a *R*^2^ value of 0.99.

### 3.4 Inclusion of Hydroxyl Group

**Figure 8:**
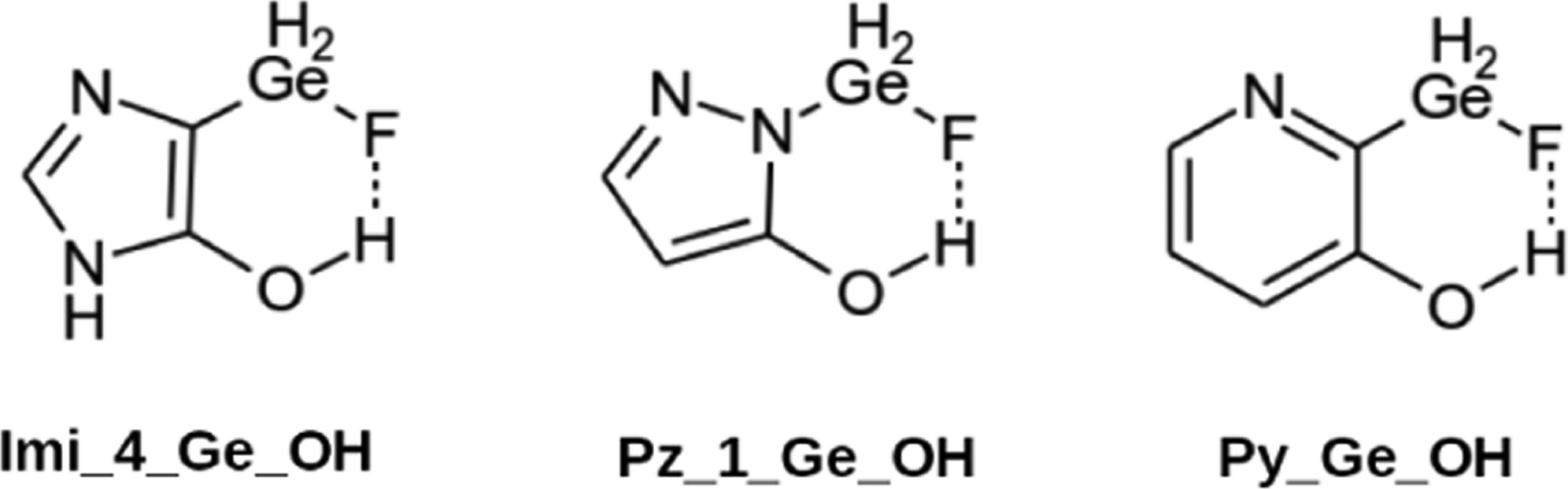
Schematic representation of the three additional FLP-OH derivatives.

An alternative method to induce a field effect in the region where the reaction between the FLP molecule and CO_2_ occurs is to modify the chemical composition of the reactants. Thus, we decided to add a hydroxyl group near the GeH_2_F group of the FLP in three molecules (Figure 8). The Cartesian coordinates of the stationary points along the reaction coordinate characterized in this section are gathered in Table S10.

**Figure 9:**
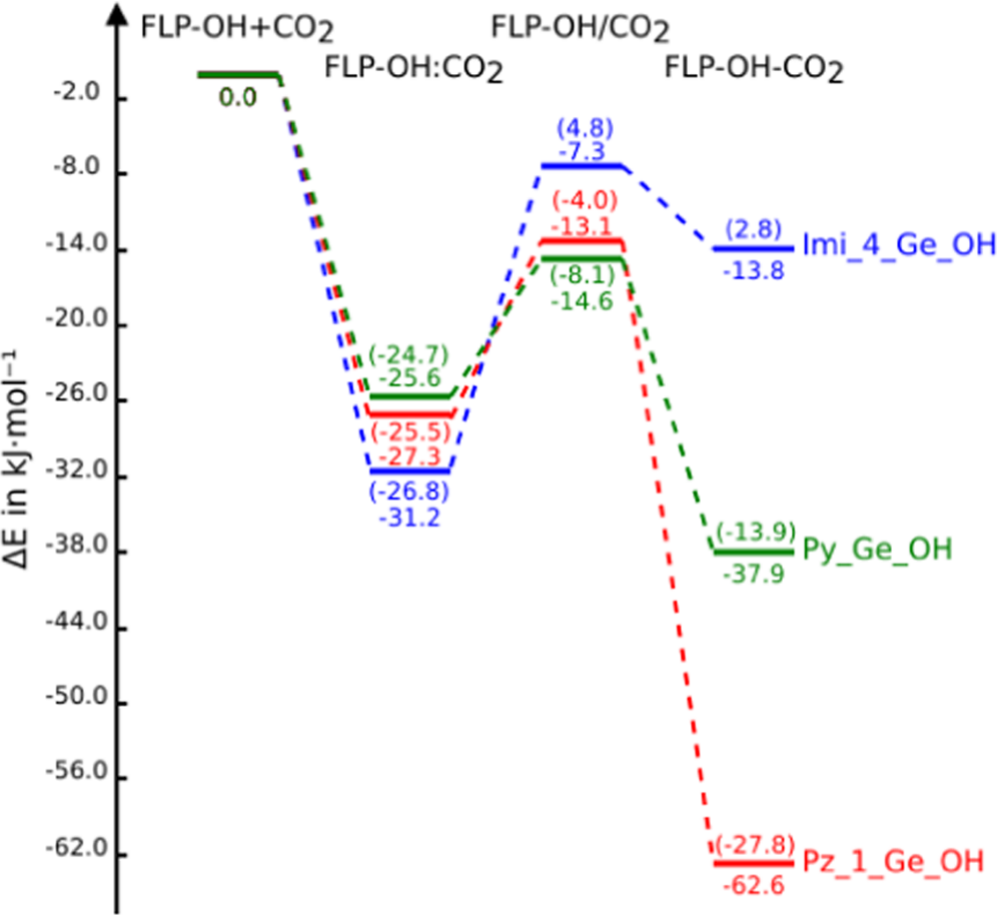
Potential energy surface of the reactions between FLP-OH + CO_2_. For comparative purposes, the values of the corresponding systems without hydroxyl group has been added in parenthesis.

The most stable configuration of the FLP-OH molecules shows a hydrogen bond between the hydroxyl group and the fluorine atom of the GeH_2_F group. The intramolecular interaction affects the MEP, increasing the σ-hole associated to the Ge–F bond (between 22 and 45 kJ mol^–1^) (Table 3). Thus, the stabilization in the complexes increases between 1 and 4 kJ mol^–1^ when compared to the analogous systems without the hydroxyl group (see Figure 9 and Table S11). Even larger effects are observed in the stabilization of the TS (between 6 and 12 kJ mol^–1^) and the adducts (between 17 and 35 kJ mol^–1^). Thus, with the inclusion of the OH group in the molecules, the adducts of the Pz_1_Ge_OH and Py_Ge_OH systems are more stable than the complexes, as opposed to the corresponding cases with no hydroxyl group.

**Table 3:** Molecular Electrostatic Potential (kJ mol^–1^) Stationary Points on the 0.001 Electron Density Isosurface of the Three Isolated FLP-OH Molecules Considered

FLP	*V*_s,min_ (N)	*V*_s,max_ (Ge–F)
Imi_4_Ge_OH	–145.6	142.6
Pz_1_Ge_OH	–114.6	143.3
Py_Ge_OH	–100.5	101.1

In analogy with the energy results, the intermolecular N···C and Ge···O distances are shorter in the three stationary points (Table S12) when the hydroxyl groups are added to the FLP molecules except for the N···C distance in the TS that are about 0.1 Å longer.

## 4 Conclusions

A theoretical study of the reaction of FLP based on nitrogen heterocycles with silane/germane groups in α to one nitrogen with CO_2_ has been carried out. The results obtained here support the following conclusions:

- The molecular electrostatic potential of the isolated FLPs and CO_2_ shows the complementarity required for the formation of the pre-reactive complexes and further the adducts.
- In the gas phase, the two minima (pre-reactive complexes FLP:CO_2_ and adducts FLP–CO_2_) are found for all of the FLPs except for the Pz_3_Ge + CO_2_ system where only the pre-reactive complex was located. The pre-reactive complexes, FLP:CO_2_, are more stable than the corresponding adducts FLP–CO_2_ except for the Pz_1_Si and Pz_1_Ge cases.
- The inclusion of the solvent effect changes the stability found in gas phase due to the large dipole moment found on the adducts that show larger solvation energies than the complexes. Relationships between the reaction energy and the barrier with the inverse of the dielectric constant have been obtained.
- The inclusion of a hydroxyl group in the heterocyclic ring of the FLPs has shown to be an alternative method to change the energy profile favoring the adducts.

## Supporting Information Available

Geometries and energies of the optimized systems; relative energy of the stationary points; γ and β parameters; electron density properties at the intermolecular bond critical points; classification of the tetrel bond contacts; NBO charge-transfer stabilization energies; electronic energy vs free energy profile; evolution of selected distances along the reaction coordinate; ρ_BCP_ vs interactomic distance; and adduct-complex energy difference vs the inverse of the dielectric constant (PDF)
