# Supplementary material for: Sequestration of Carbon Dioxide with Frustrated Lewis Pairs Based on N-Heterocycles with Silane/Germane Groups
Source: J Phys Chem A. 2021 Aug 10;125(32):6976–84. doi: 10.1021/acs.jpca.1c04787 (PMC8389994; doi:10.1021/acs.jpca.1c04787)
Supplement: Supplementary file 1 — jp1c04787_si_001.pdf [file jp1c04787_si_001.pdf]

Supporting Information

Sequestration of Carbon Dioxide with FLP Based on N-heterocycles with Silane/Germane Groups.

Maxime Ferrer,[a,b] Ibon Alkorta,[a,]* José Elguero[a], Josep M. Oliva-Enrich[c]

[a]Instituto de Química Médica (CSIC), Juan de la Cierva, 3, E-28006 Madrid, Spain

[b] PhD Programme in Theoretical Chemistry and Computational Modelling, Doctoral School, Universidad Autónoma de Madrid, Spain.

[c] Instituto de Química-Física Rocasolano (CSIC), Serrano, 119, E-28006 Madrid, Spain

Index

Pg. S3-21 Table S1. Geometries, energies (hartree) and Cartesian coordinates (Å) the systems optimized at the MP2/Jul-cc-pVTZ computational level.

Pg. S22 Table S2. Relative energy (kJ mol^{-1}) of the stationary points in the reaction with respect to the isolated FLP + CO_2

Pg. S23 Fig. S1. Electronic energy vs. Free energy profile (kJ/mol).

Pg. S24 Fig. S2. Evolution of the N-C, O-Si/Ge, C-O(1), and C-O(2) distances along the reaction coordinate.

Pg.S25 Table S3. γ and β parameters (see Eq. 1 and Eq. 2 of the main text).

Pg. S26-27 Table S4. Electron density properties (au) at the intermolecular BCP's calculated in vacuo.

Pg. S28 Table S5. Classification of the tetrel bonds contacts based on the BCP properties

Pg. S29 Fig. S3. ρ_{BCP} (au) vs. interactomic distance (Å) in the C-N, Si-O and Ge-O contacts along the reaction coordinate.

Pg. S30-31 Table S6. NBO charge transfer stabilization energies

Pg. S32 Table S7. Relative energy (kJ mol^{-1}) of the stationary points in the reaction with respect to the isolated FLP + CO_2 including the effect of the solvent [PCM(Acetonitrile)].

Pg. S33 Table S8. γ and β parameters with the PCM(acetonitrile) model

Pg. S34-35 Table S9. Electron density properties (au) at the intermolecular BCP's calculated using the PCM(acetonitrile) model.

Pg. S28 Fig. S4 Adduct-Complex energy difference (kJ mol^{-1}) for the Imi_2_Ge + CO_2 reaction as a function of the inverse of the dielectric constant of the solvent.

Pg. S37-42 Table S10. Geometries, energies (hartree) and Cartesian coordinates (Å) the systems with a hydroxyl group optimized at the MP2/Jul-cc-pVTZ computational level.

Pg. S43 Table S11. Relative energies (kJ mol^{-1}) of the stationary points with the inclusion of a hydroxyl group.

Pg. S44 Table S12. Selected geometrical parameters (Å) of the stationary points with the inclusion of a hydroxyl group.

Table S1.Geometries, energies (hartree) and Cartesian coordinates (Å) the molecules.

**MONOMERS (VACUUM)**	
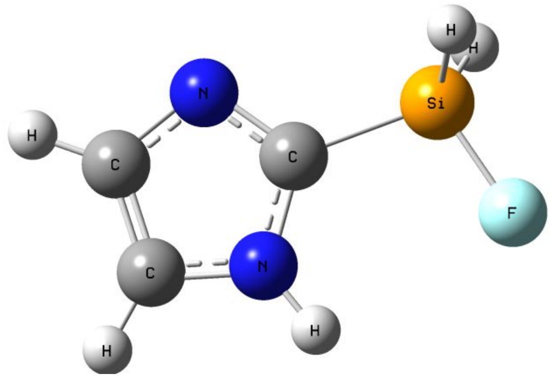	**Imi_2_SiH2F**<br>EMP2= -615.24903 NIMAG=0<br>N,0.2881816738,1.0415851772,0.<br>C,-0.7985557448,0.2611771041,0.<br>N,-1.9298921666,1.0345803945,0.<br>C,-1.5535518271,2.347832378,0.<br>C,-0.1729726249,2.3281673383,0.<br>H,-2.2683428379,3.1519720554,0.<br>H,-2.8754091347,0.6840583028,0.<br>H,0.4981150969,3.1705509065,0.<br>Si,-0.7642571246,-1.594510019,0.<br>F,-2.3266755975,-2.0331368942,0.<br>H,-0.1316487,-2.1390607,-1.2129901<br>H,-0.1316487,-2.1390607,1.2129901
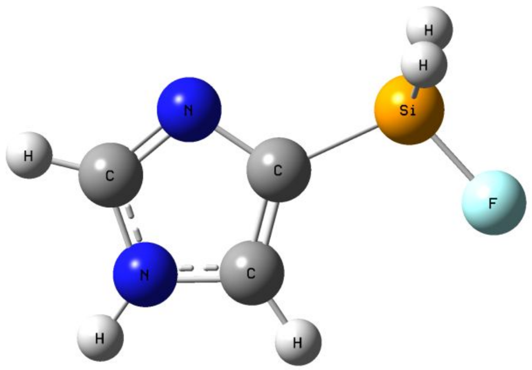	**Imi_4_SiH2F**<br>EMP2= -615.24882 NIMAG=0<br>C,-0.1471688397,2.2410362773,0.<br>N,0.253154905,0.9828016145,0.<br>C,-0.9068985818,0.2209681939,0.<br>C,-2.0085416375,1.0564488428,0.<br>N,-1.5071821801,2.3302319175,0.<br>H,-2.0458705522,3.181148266,0.<br>H,-3.0657138881,0.8541674074,0.<br>H,0.4898425461,3.1098845286,0.<br>Si,-0.8239660686,-1.6286829097,0.<br>F,-2.3523500733,-2.1672947277,0.<br>H,-0.1609153,-2.1497962,-1.2098133<br>H,-0.1609153,-2.1497962,1.2098133
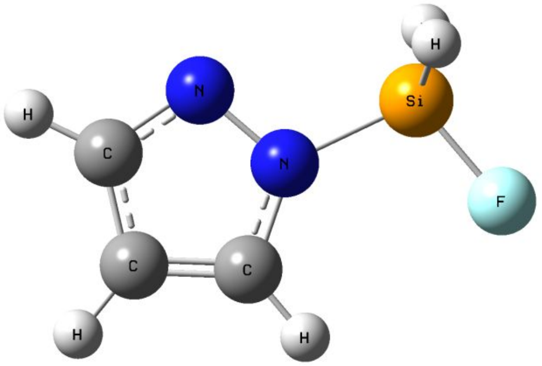	**Pz_1_SiH2F**<br>EMP2= -615.25021 NIMAG=0<br>C,-0.138342,2.168278,0.000000<br>N,0.197848,0.869187,0.000000<br>N,-0.988041,0.204829,0.000000<br>C,-2.052240,1.059406,0.000000<br>C,-1.533117,2.339817,0.000000<br>H,-2.087752,3.261908,0.000000<br>H,-3.064241,0.690186,0.000000<br>H,0.639043,2.914516,0.000000<br>Si,-0.814050,-1.550483,0.000000<br>F,-2.321505,-2.108282,0.000000

	F,-0.137065,-1.984122,-1.226878<br>F,-0.137065,-1.984122,1.226878
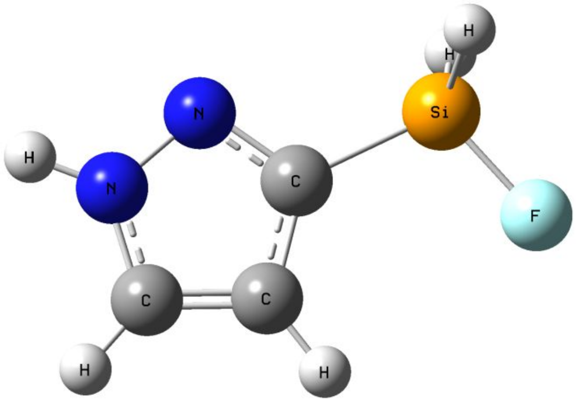	**Pz_3_SiH2F**<br>EMP2= -615.23124 NIMAG=0<br>N,-0.1871383261,2.2332327504,0.<br>N,0.2312446319,0.9617158346,0.<br>C,-0.9124726691,0.2305053701,0.<br>C,-2.0418047198,1.0760939808,0.<br>C,-1.5370415987,2.3643904545,0.<br>H,-2.0108521986,3.331116373,0.<br>H,-3.0786988787,0.7871307267,0.<br>H,0.5011354755,2.9692014968,0.<br>Si,-0.8030412466,-1.6235210043,0.<br>F,-2.3253534389,-2.1726455798,0.<br>H,-0.1362510,-2.13805168,-1.2104245326<br>H,-0.1362510,-2.13805168,1.2104245326
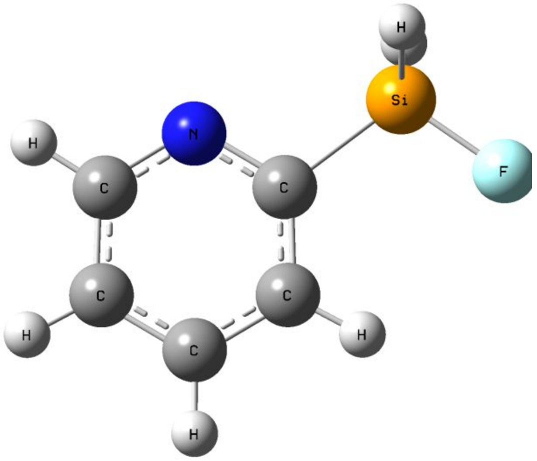	**Py_SiH2F**<br>EMP2= -637.22785 NIMAG=0<br>N,-0.045956,-0.178414,0.000000<br>C,-0.711629,-1.340129,0.000000<br>C,-0.772778,0.964251,0.000000<br>C,-2.103239,-1.425274,0.000000<br>C,-2.169895,0.965552,0.000000<br>C,-2.848098,-0.250494,0.000000<br>H,-0.104106,-2.237669,0.000000<br>H,-2.585367,-2.393735,0.000000<br>H,-2.707415,1.905491,0.000000<br>H,-3.929947,-0.282262,0.000000<br>Si,0.317159,2.487202,0.000000<br>F,-0.647406,3.789101,0.000000<br>H,1.150847,2.535921,1.214742<br>H,1.150847,2.535921,-1.214742
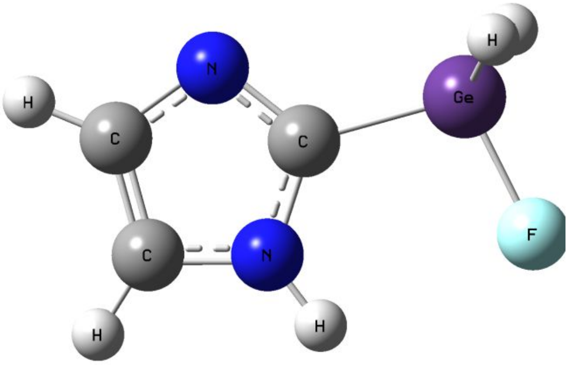	**Imi_2_GeH2F**<br>EMP2= -2401.76667 NIMAG=0<br>N,0.334577,1.110757,0.015558<br>C,-0.721809,0.295749,0.010183<br>N,-1.882759,1.01673,-0.004798<br>C,-1.558271,2.344754,-0.009201<br>C,-0.178519,2.379505,0.003619<br>H,-2.303819,3.120128,-0.020754<br>H,-2.809739,0.618597,-0.01101<br>H,0.458639,3.247695,0.004716<br>Ge,-0.668061,-1.610807,0.021694<br>F,-2.374581,-1.997015,0.007561

	H,-0.096426,-2.197192,1.293056<br>H,-0.072143,-2.211801,-1.231576
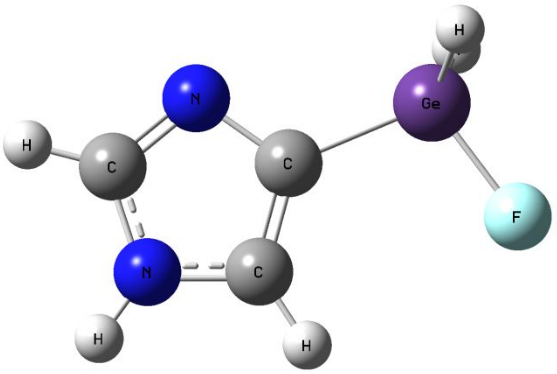	**Imi_4_GeH2F**<br>EMP2= -2401.76620 NIMAG=0<br>C,-0.152353,2.301057,-0.000385<br>N,0.296673,1.057927,0.005548<br>C,-0.832668,0.259039,0.006043<br>C,-1.967034,1.045059,0.000264<br>N,-1.514164,2.337327,-0.003711<br>H,-2.084386,3.167471,-0.00873<br>H,-3.013801,0.795557,-0.001579<br>H,0.450837,3.193546,-0.002553<br>Ge,-0.720816,-1.64178,0.013558<br>F,-2.38666,-2.163991,-0.005423<br>H,-0.110972,-2.203965,1.281143<br>H,-0.078247,-2.210479,-1.234696
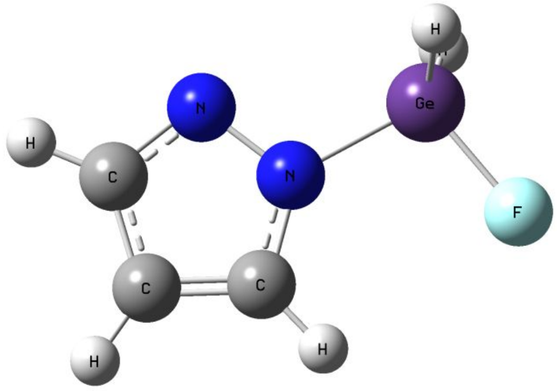	**Pz_1_GeH2F**<br>EMP2= -2401.75950 NIMAG=0<br>C,-0.135533,2.209311,-0.000478<br>N,0.232083,0.91605,0.003344<br>N,-0.935578,0.228572,0.003272<br>C,-2.020678,1.051802,-0.000185<br>C,-1.532245,2.346361,-0.003201<br>H,-2.109472,3.25446,-0.007092<br>H,-3.021882,0.654111,-0.001092<br>H,0.624256,2.973384,-0.001617<br>Ge,-0.744952,-1.606393,0.015805<br>F,-2.38786,-2.145423,-0.008151<br>H,-0.12105,-2.049096,1.314295<br>H,-0.076629,-2.059659,-1.2564
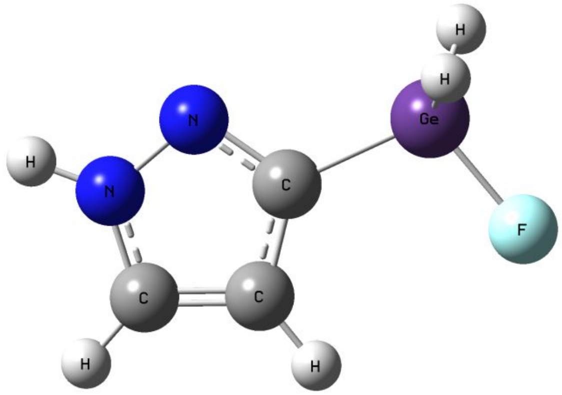	**Pz_3_GeH2F**<br>EMP2= -2401.74863 NIMAG=0<br>N,-0.168293,2.255768,-0.001704<br>N,0.26969,0.989468,0.002529<br>C,-0.863197,0.247278,0.004677<br>C,-2.005852,1.069428,0.001611<br>C,-1.519909,2.365156,-0.002494<br>H,-2.008871,3.324187,-0.006049<br>H,-3.03627,0.758649,0.001917<br>H,0.508576,3.002138,-0.004248<br>Ge,-0.742597,-1.658605,0.012057<br>F,-2.404384,-2.184953,-0.009572<br>H,-0.134041,-2.217217,1.281588<br>H,-0.09695,-2.223716,-1.235952

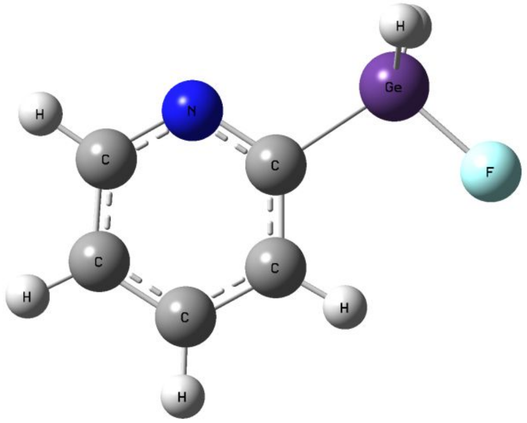	**Py_GeH2F**<br>EMP2= -2423.74666 NIMAG=0<br>N,-0.035594,-0.248,0.003523<br>C,-0.732508,-1.392257,0.000741<br>C,-0.735595,0.906495,0.003629<br>C,-2.125328,-1.441295,-0.001986<br>C,-2.129874,0.950622,0.001051<br>C,-2.839127,-0.247376,-0.001873<br>H,-0.148429,-2.305054,0.000741<br>H,-2.632093,-2.397005,-0.004208<br>H,-2.636834,1.907074,0.001062<br>H,-3.921359,-0.250076,-0.004013<br>Ge,0.381918,2.474873,0.008451<br>F,-0.729729,3.821863,-0.004445<br>H,1.199328,2.601503,1.276601<br>H,1.220725,2.593222,-1.246324

COMPLEXES (VACUUM)	
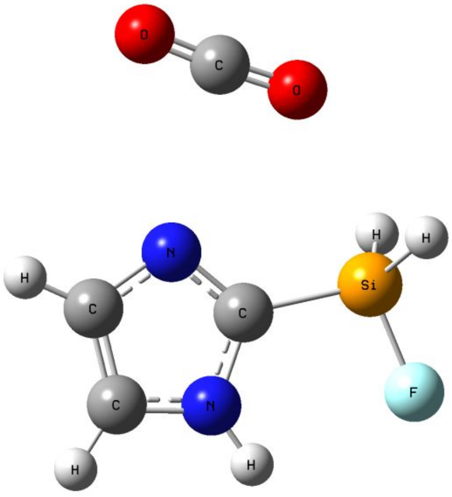	**Imi_2_SiH2F:CO2**<br>EMP2= -803.58051 NIMAG=0<br>C,2.7162192174,-0.152944268,0.<br>O,2.1252686283,-1.1673786677,0.<br>O,3.3738297775,0.8111497116,0.<br>N,0.3288736433,1.0966940147,0.<br>C,-0.7228910616,0.267590325,0.<br>N,-1.8820336105,1.0007388284,0.<br>C,-1.5611067851,2.3272285881,0.<br>C,-0.1809963676,2.3637758716,0.<br>H,-2.3076692297,3.1019530349,0.<br>H,-2.8109905803,0.6070667921,0.<br>H,0.4587388287,3.2304229343,0.<br>Si,-0.7509680212,-1.5974338114,0.<br>F,-2.3526631719,-1.8957652184,0.<br>H,-0.19247567,-2.19405806,-1.2217164<br>H,-0.19247567,-2.1940581,1.22171640
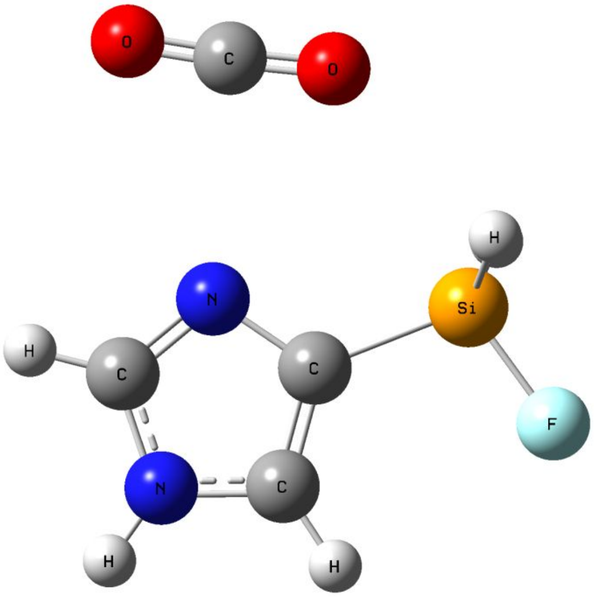	**Imi_4_SiH2F:CO2**<br>EMP2= -803.57935 NIMAG=0<br>C,2.7743828228,-0.0852833944,0.<br>O,2.2592378378,-1.1381862426,0.<br>O,3.3513106883,0.9311289405,0.<br>C,-0.153450332,2.2783286765,0.<br>N,0.289614462,1.0340203222,0.<br>C,-0.838754283,0.2266640272,0.<br>C,-1.9684314769,1.0257880849,0.<br>N,-1.5144321034,2.3169706273,0.<br>H,-2.0836822389,3.147923174,0.<br>H,-3.0172217365,0.783687323,0.<br>H,0.4555929212,3.1671791508,0.<br>Si,-0.7944929774,-1.6316065991,0.<br>F,-2.3562221123,-2.0754547163,0.<br>H,-0.17739578,-2.18808868,-1.2158926<br>H,-0.17739578,-2.18808868,1.2158926

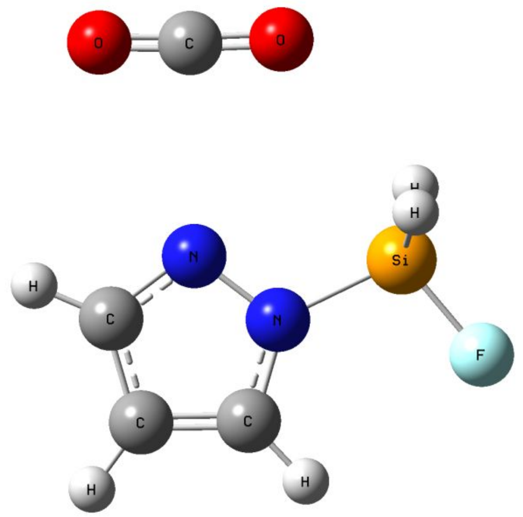

Pz_1_SiH2F:CO2
EMP2= -803.57999   NIMAG=0
C,2.8120704159,-0.0844695093,0.
O,2.4201443947,-1.1892035294,0.
O,3.2529701122,0.9975380102,0.
C,-0.1395850351,2.1906220315,0.
N,0.2196436592,0.8974675207,0.
N,-0.9498588606,0.2068880795,0.
C,-2.029651301,1.0432030883,0.
C,-1.5365984196,2.333946896,0.
H,-2.1097385235,3.2446049611,0.
H,-3.0343312159,0.6544127251,0.
H,0.6267014954,2.9487794624,0.
Si,-0.8146684256,-1.5592250398,0.
F,-2.344604612,-2.0547850776,0.
H,-0.16191688,-2.01239880,-1.2314048
H,-0.16191688,-2.0123988,1.23140484

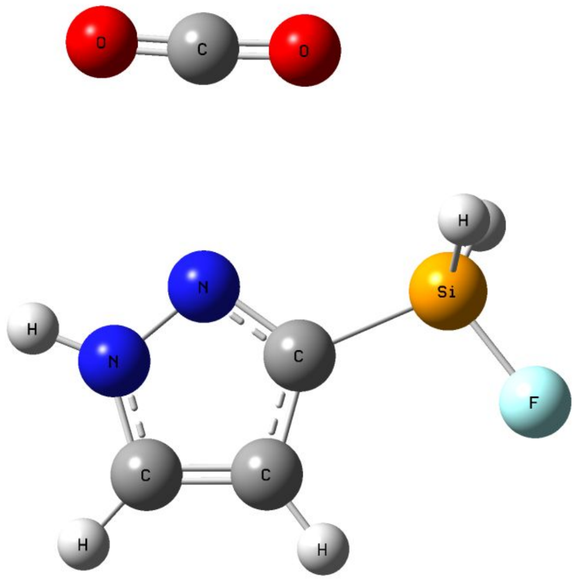

Pz_3_SiH2F:CO2
EMP2= -803.56137   NIMAG=0
C,2.7998116568,-0.0214293137,0.
O,2.3571242977,-1.1054713926,0.
O,3.2945349449,1.0386245643,0.
N,-0.1681008257,2.2254707874,0.
N,0.2535049323,0.9562082178,0.
C,-0.8831331559,0.2144401671,0.
C,-2.0155454405,1.0566004089,0.
C,-1.5186703353,2.3481727121,0.
H,-1.9983271377,3.3120232545,0.
H,-3.0506044784,0.7609780706,0.
H,0.5196054923,2.9629186635,0.
Si,-0.8155162256,-1.6469948548,0.
F,-2.3639557809,-2.1235286699,0.
H,-0.18103401,-2.18651530,-1.2150238
H,-0.18103401,-2.1865153,1.21502384

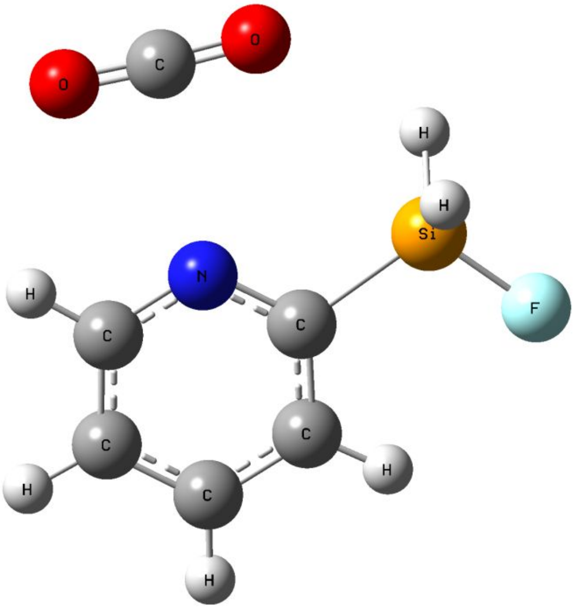	**Py_SiH2F:CO2**<br>EMP2= -825.55784 NIMAG=0<br>C,2.6949410335,-0.6380951173,0.<br>O,2.518545695,-1.7949579146,0.<br>O,2.9357284719,0.5079130293,0.<br>N,-0.0363357509,-0.2181157617,0.<br>C,-0.7219848139,-1.3684385658,0.<br>C,-0.7364969572,0.9418335643,0.<br>C,-2.1144961154,-1.4279855863,0.<br>C,-2.1345155332,0.9638652275,0.<br>C,-2.8357925628,-0.2390664922,0.<br>H,-0.1254747448,-2.2736026152,0.<br>H,-2.61452739,-2.3872049053,0.<br>H,-2.6558248619,1.9127777231,0.<br>H,-3.9180744498,-0.2489056454,0.<br>Si,0.3192349938,2.4964001352,0.<br>F,-0.7150839397,3.7451653253,0.<br>H,1.14119462,2.58936973,1.21860294<br>H,1.14119462,2.58936973,-1.21860294
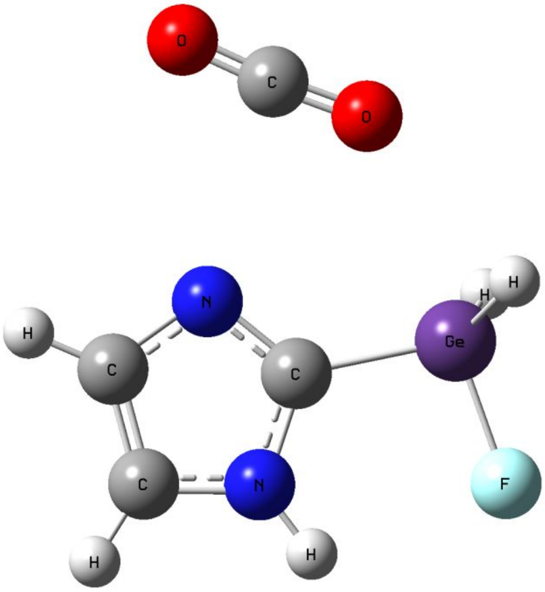	**Imi_2_GeH2F:CO2**<br>EMP2= -2590.09984 NIMAG=0<br>C,2.6959753468,-0.1527648361,0.<br>O,2.0758038794,-1.1509696074,0.<br>O,3.3872391695,0.7860742632,0.<br>N,0.3548332434,1.1461783973,0.<br>C,-0.6781555184,0.2991907599,0.<br>N,-1.8539883964,0.9957624185,0.<br>C,-1.5659246227,2.3308113652,0.<br>C,-0.1875768016,2.4018116405,0.<br>H,-2.3311029317,3.0869594897,0.<br>H,-2.7674766589,0.5658078926,0.<br>H,0.429709291,3.2844549698,0.<br>Ge,-0.6894824585,-1.6169722263,0.<br>F,-2.4332302498,-1.8483525432,0.<br>H,-0.19398169,-2.26150498,-1.2717887<br>H,-0.19398169,-2.26150498,1.2717887

<table>
<tr>
<td>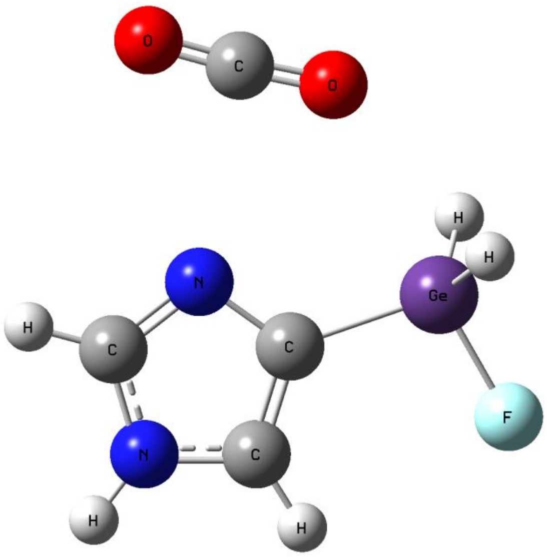</td>
<td>Imi_4_GeH2F:CO2
EMP2= -2590.09806   NIMAG=0
C,2.7388646314,-0.104936569,0.
O,2.1648934286,-1.1282843879,0.
O,3.3772245772,0.8726058971,0.
C,-0.1639885425,2.3326008995,0.
N,0.3194513857,1.1019021284,0.
C,-0.7817396165,0.2650852149,0.
C,-1.937606034,1.0211691114,0.
N,-1.5247535185,2.3264998666,0.
H,-2.1201042889,3.1389864942,0.
H,-2.9756561219,0.7377460572,0.
H,0.4155072999,3.2408053218,0.
Ge,-0.7193419412,-1.6457751665,0.
F,-2.4253381957,-2.0423071394,0.
H,-0.15937657,-2.2555579,-1.26548446
H,-0.15937657,-2.2555579,1.26548446</td>
</tr>
<tr>
<td>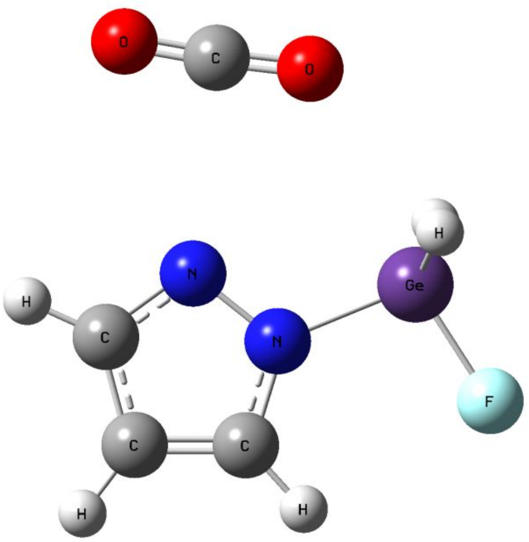</td>
<td>Pz_1_GeH2F:CO2
EMP2= -2590.09084   NIMAG=0
C,2.7469205492,-0.1225305638,0.
O,2.253402531,-1.1878795829,0.
O,3.297573914,0.9058862163,0.
C,-0.1537111298,2.2709956091,0.
N,0.2650009029,0.9932404019,0.
N,-0.8691084058,0.2539233045,0.
C,-1.9864428595,1.0356606679,0.
C,-1.5537288125,2.349648812,0.
H,-2.1688843572,3.2324972808,0.
H,-2.9702251863,0.5967942502,0.
H,0.5771668436,3.0632009906,0.
Ge,-0.7267234848,-1.5952139635,0.
F,-2.4035664166,-2.0302198012,0.
H,-0.12950708,-2.08051081,-1.2932939
H,-0.12950708,-2.08051081,1.2932939</td>
</tr>
</table>

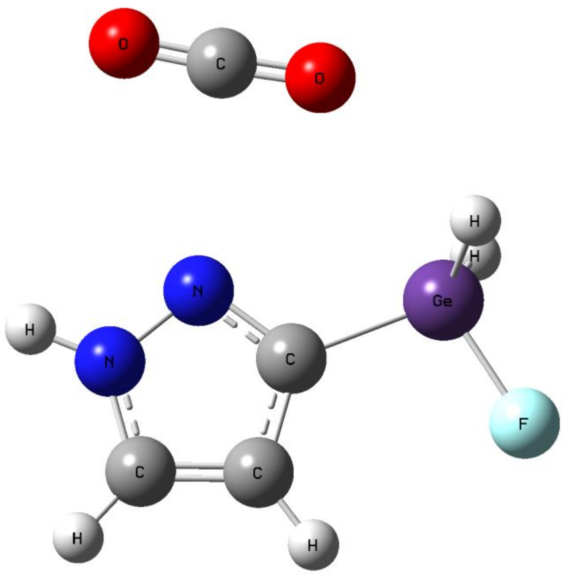	**Pz_3_GeH2F:CO2**<br>EMP2= -2590.07977 NIMAG=0<br>C,2.7657623672,-0.0693039954,0.<br>O,2.2351278285,-1.1144848086,0.<br>O,3.3488090088,0.943208272,0.<br>N,-0.1878098675,2.292440065,0.<br>N,0.2829015039,1.0389965785,0.<br>C,-0.8252507804,0.2602825738,0.<br>C,-1.9892381056,1.053023269,0.<br>C,-1.5419363413,2.3625103669,0.<br>H,-2.0584500108,3.3070599461,0.<br>H,-3.0095953651,0.7106459401,0.<br>H,0.4701183013,3.0561997405,0.<br>Ge,-0.7291358922,-1.653169292,0.<br>F,-2.4190051929,-2.0979255543,0.<br>H,-0.14681877,-2.24225054,-1.2652950<br>H,-0.14681877,-2.24225054,1.2652950
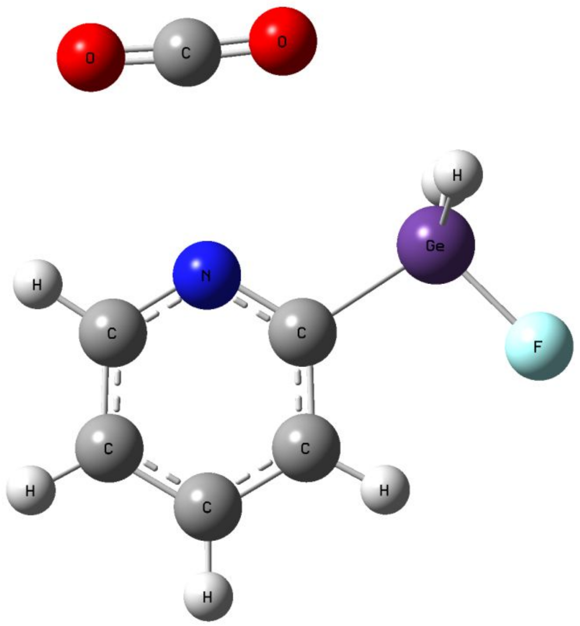	**Py_GeH2F:CO2**<br>EMP2= -2612.07770 NIMAG=0<br>C,2.6558347739,-0.5895461396,0.<br>O,2.5713631734,-1.7555603439,0.<br>O,2.8145634088,0.572089605,0.<br>N,-0.0304840609,-0.2987792083,0.<br>C,-0.7507245051,-1.4283615733,0.<br>C,-0.6956525039,0.8760879971,0.<br>C,-2.1436821517,-1.4457159712,0.<br>C,-2.0902113046,0.9467340573,0.<br>C,-2.8271953799,-0.2345155021,0.<br>H,-0.1817138879,-2.3512355288,0.<br>H,-2.6728876636,-2.3890719513,0.<br>H,-2.5762289554,1.9137339423,0.<br>H,-3.909177667,-0.2101786464,0.<br>Ge,0.3916458565,2.4759916829,0.<br>F,-0.8042663125,3.7530647977,0.<br>H,1.19552475,2.65779232,1.26773564<br>H,1.19552475,2.65779232,-1.26773564

ADDUCTS (VACUUM)	
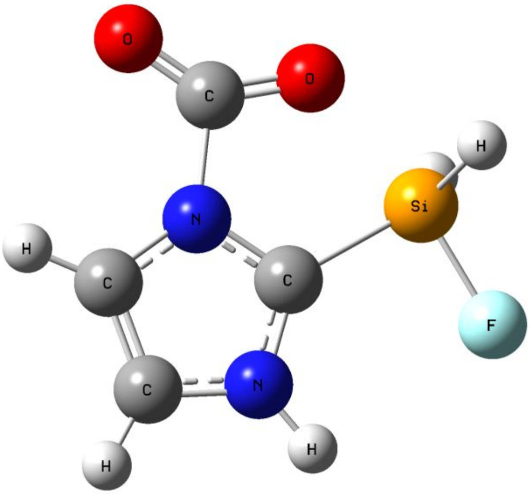	**Imi_2_SiH2F-CO2**<br>EMP2= -803.57169 NIMAG=0<br>C,1.3900826824,0.5395761649,0.<br>O,1.8671378531,-0.6335146462,0.<br>O,1.8495949312,1.6552284252,0.<br>N,-0.1272181222,0.4245028049,0.<br>C,-0.607851656,-0.8195545564,0.<br>N,-1.946616841,-0.6939485823,0.<br>C,-2.302724518,0.6327111552,0.<br>C,-1.1287078403,1.3497157373,0.<br>H,-3.327260605,0.9592958128,0.<br>H,-2.5676159275,-1.4910253028,0.<br>H,-0.935300765,2.407632007,0.<br>Si,0.639296987,-2.2564822601,0.<br>H,1.27243923,-2.59714375,1.28052246<br>H,1.27243923,-2.59714375,-1.2805225<br>F,-0.5962629277,-3.3880647254,0.
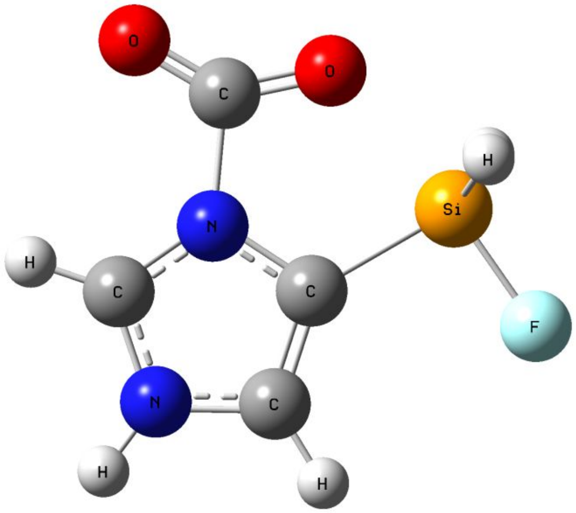	**Imi_4_SiH2F-CO2**<br>EMP2= -803.56909 NIMAG=0<br>C,1.4025894158,0.5696310555,0.<br>O,1.9042966751,-0.577891909,0.<br>O,1.7927912781,1.7147396903,0.<br>C,-1.0950689145,1.3063705048,0.<br>N,-0.1320913216,0.3953171184,0.<br>C,-0.6370773505,-0.8765227304,0.<br>C,-2.0063799463,-0.7240705554,0.<br>N,-2.2599191563,0.6282135445,0.<br>H,-3.1717507728,1.0601966519,0.<br>H,-2.7948185773,-1.4554507343,0.<br>H,-0.9621707404,2.3736384,0.<br>Si,0.6248895393,-2.2943784152,0.<br>H,1.30170868,-2.5650568,1.27455051<br>H,1.30170868,-2.5650568,-1.27455051<br>F,-0.5172757776,-3.4978944843,0.

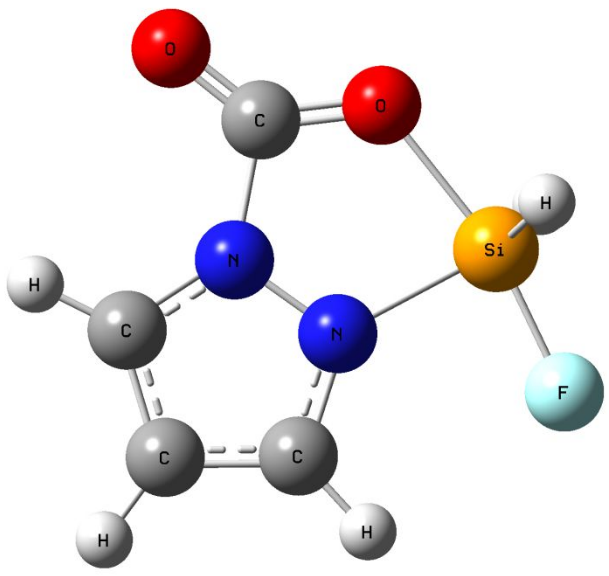	**Pz_1_SiH2F-CO2**<br>EMP2= -803.58107 NIMAG=0<br>C,1.3662460155,0.5250182897,0.<br>O,1.8293741191,-0.6626681537,0.<br>O,1.8577041088,1.6227769592,0.<br>C,-1.1192938806,1.3332576995,0.<br>N,-0.1256737468,0.427950817,0.<br>N,-0.6052774471,-0.8122839493,0.<br>C,-1.9513572923,-0.7324382603,0.<br>C,-2.3117010003,0.615117995,0.<br>H,-3.308527784,1.0194080689,0.<br>H,-2.5375538,-1.634744409,0.<br>H,-0.8925806718,2.3855909329,0.<br>Si,0.6655956424,-2.1985950547,0.<br>H,1.2457797,-2.56601795,1.29648401<br>H,1.2457797,-2.56601795,-1.29648401<br>F,-0.6070819635,-3.2645705049,0.
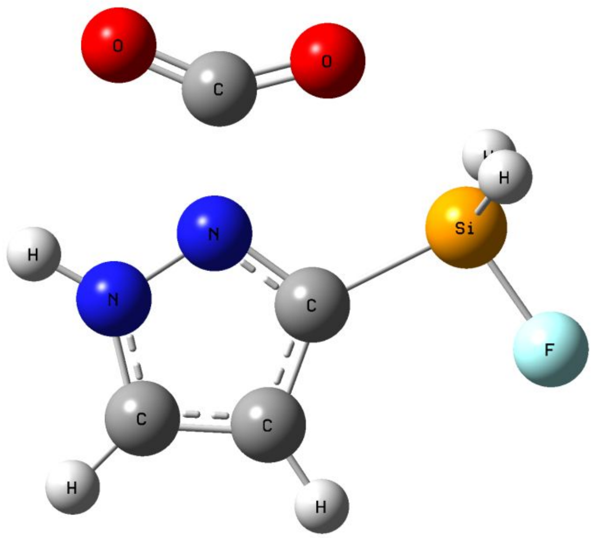	**Pz_3_SiH2F-CO2**<br>EMP2= -803.54408 NIMAG=0<br>C,1.4385014331,0.6421076518,0.<br>O,2.0049275795,-0.4513058487,0.<br>O,1.6576264855,1.8342608077,0.<br>N,-1.0739186169,1.2443218898,0.<br>N,-0.1237934698,0.3289755447,0.<br>C,-0.6486263595,-0.9034429262,0.<br>C,-2.0447335689,-0.7331865161,0.<br>C,-2.2848365201,0.6358144755,0.<br>H,-3.1943067238,1.2116313871,0.<br>H,-2.7849602194,-1.5141999666,0.<br>H,-0.7907675245,2.2185117617,0.<br>Si,0.558324902,-2.3621047146,0.<br>H,1.28831633,-2.54030245,1.25880956<br>H,1.28831631,-2.54030245,-1.2588096<br>F,-0.5386383473,-3.5789941151,0.

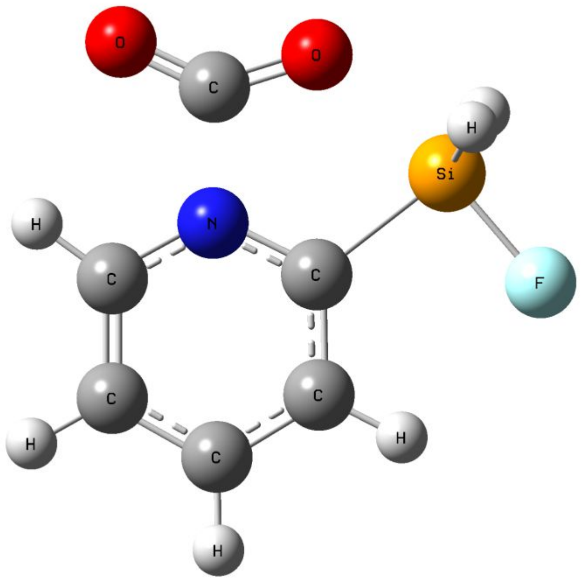	**Py_SiH2F-CO2**<br>EMP2= -825.55623 NIMAG=0<br>C,1.6406372775,-0.9036785633,0.<br>O,1.8572762936,-2.0902853785,0.<br>O,2.316622742,0.1491723439,0.<br>N,0.1134326346,-0.5101960203,0.<br>C,-0.8169864396,-1.4722018728,0.<br>C,-0.1440027293,0.809367479,0.<br>C,-2.1547752335,-1.1122596333,0.<br>C,-1.4834161648,1.1994171392,0.<br>C,-2.4920072583,0.2397260409,0.<br>H,-0.4356634932,-2.4842196113,0.<br>H,-2.9121961646,-1.8827371519,0.<br>H,-1.7053527936,2.2572579596,0.<br>H,-3.531640933,0.5394954724,0.<br>Si,1.3901754805,1.9676899886,0.<br>F,0.4556279251,3.3485786114,0.<br>H,2.0832001,2.15854214,1.28231466<br>H,2.0832001,2.15854214,-1.28231466
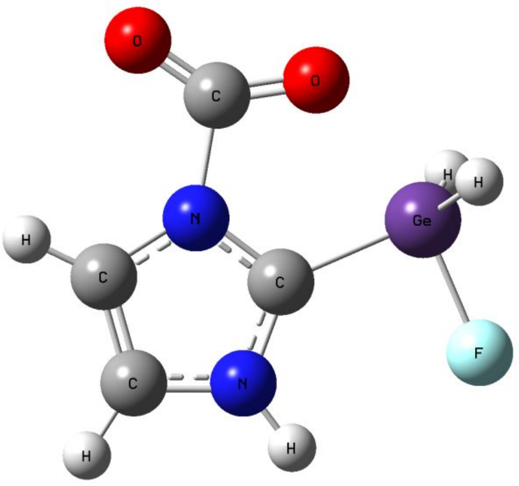	**Imi_2_GeH2F-CO2**<br>EMP2= -2590.09418 NIMAG=0<br>C,1.4242962399,0.6054715776,0.<br>O,1.9432227981,-0.5393570199,0.<br>O,1.814283265,1.748656238,0.<br>N,-0.1132914327,0.4383833405,0.<br>C,-0.5891102811,-0.8049378401,0.<br>N,-1.9286232385,-0.6985063383,0.<br>C,-2.2962906115,0.6243433498,0.<br>C,-1.127705011,1.3503948948,0.<br>H,-3.3235535614,0.9420186785,0.<br>H,-2.5342143548,-1.5080806976,0.<br>H,-0.9434599385,2.4099822662,0.<br>Ge,0.63438671,-2.30828899,0.<br>H,1.27625758,-2.65961715,1.31504608<br>H,1.27625758,-2.65961715,-1.3150461<br>F,-0.7610240373,-3.4490606261,0.
	**Imi_4_GeH2F-CO2**<br>EMP2= -2590.08677 NIMAG=0<br>C,1.4467404974,0.6445242493,0.<br>O,1.9889918182,-0.4721481787,0.<br>O,1.7536565181,1.8148208067,0.<br>C,-1.0935183772,1.3071222997,0.<br>N,-0.1191461789,0.4084673181,0.<br>C,-0.6231730373,-0.8618435074,0.

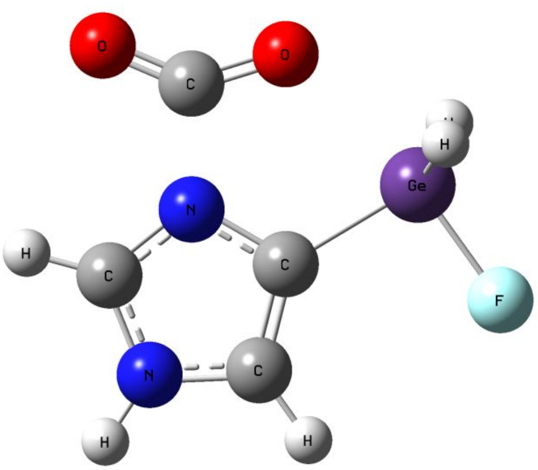

C,-1.9923478428,-0.726359566,0.
N,-2.2555983947,0.6235176578,0.
H,-3.170208054,1.0494761485,0.
H,-2.7705252799,-1.4686268811,0.
H,-0.9679320857,2.3754019821,0.
Ge,0.6094248466,-2.3543431336,0.
H,1.3066193,-2.62746670,1.30646239
H,1.3066193,-2.6274667,-1.30646239
F,-0.6681713718,-3.5932912525,0.

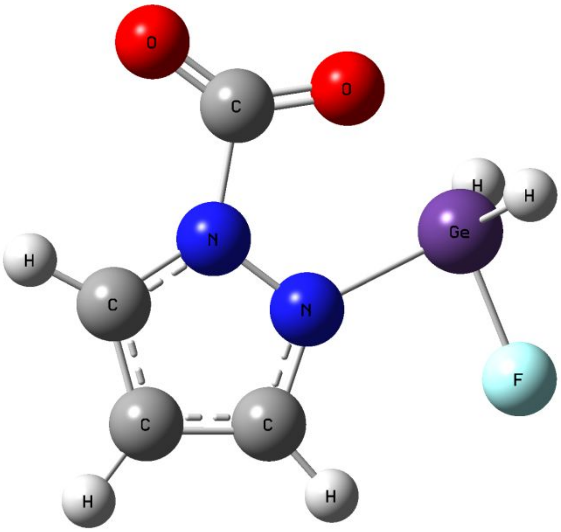

Pz_1_GeH2F-CO2
EMP2= -2590.09174   NIMAG=0
C,1.3793386736,0.5734024259,0.
O,1.8902327027,-0.5882224224,0.
O,1.8157119425,1.6961711931,0.
C,-1.1169732573,1.3382509895,0.
N,-0.1206264443,0.434117765,0.
N,-0.6032459827,-0.8036808787,0.
C,-1.9486954613,-0.7262668747,0.
C,-2.3092283451,0.6214980646,0.
H,-3.3061781196,1.0255437,0.
H,-2.5311411246,-1.6312581646,0.
H,-0.8888131328,2.3902266014,0.
Ge,0.6887873122,-2.2574422016,0.
H,1.26326174,-2.61619285,1.34335113
H,1.26326174,-2.61619285,-1.3433511
F,-0.7242605567,-3.348169953,0.

Py_GeH2F-CO2
EMP2= -2612.07359   NIMAH=0
C,1.6812454287,-0.9754720998,0.
O,1.8215091429,-2.1731363355,0.
O,2.3813828437,0.0479508049,0.
N,0.1214269443,-0.5247411524,0.
C,-0.8209876406,-1.474818336,0.
C,-0.1406464436,0.7914333666,0.
C,-2.157914263,-1.1101013374,0.
C,-1.4746030281,1.1956038985,0.
C,-2.4891922804,0.242643275,0.
H,-0.4485986667,-2.4902952819,0.
H,-2.9182168803,-1.8777973306,0.
H,-1.6847249897,2.256060741,0.
H,-3.5267350045,0.5492568973,0.

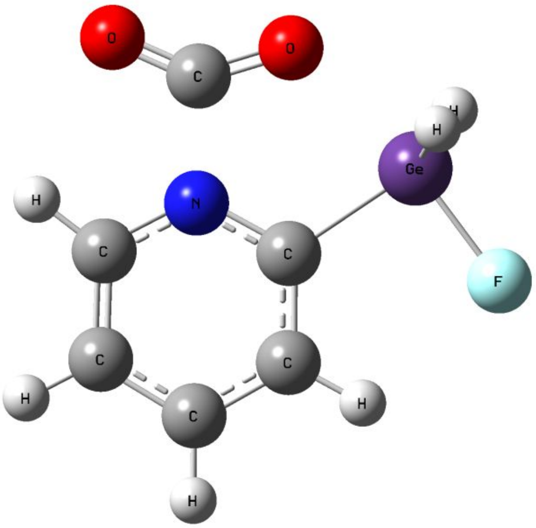	Ge,1.3865282001,2.0209063016,0.<br>F,0.3347988086,3.4698314596,0.<br>H,2.09942961,2.2124431,1.31369822<br>H,2.09942961,2.2124431,-1.31369822

TRANSITION STATES (VACUUM)	
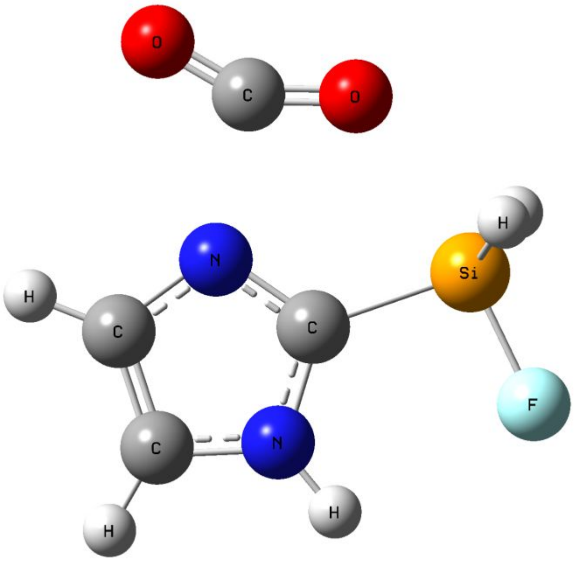	**Imi_2_SiH2F/CO2**<br>EMP2=-803.57169 NIMAG=1<br>C,1.60085513,-1.95308942,-0.0020947<br>O,0.4637165,-2.37025681,-0.0011254<br>O,2.77010829,-2.1414279,-0.00324784<br>N,1.2835752,-0.08702228,-0.00131765<br>C,-0.01241540,0.22675627,0.0003977<br>N,-0.10468308,1.58310572,0.00088462<br>C,1.14923828,2.12806306,-0.00056178<br>C,2.02048323,1.05848827,-0.00194194<br>H,1.31943478,3.1903659,-0.00050639<br>H,-0.98261439,2.08205962,0.00215875<br>H,3.09699952,1.04727258,-0.00329197<br>Si,-1.44862763,-0.98330314,0.0021007<br>F,-2.64691323,0.146071,0.004112585<br>H,-1.65190572,-1.72252985,-1.247325<br>H,-1.64823911,-1.72316425,1.251758
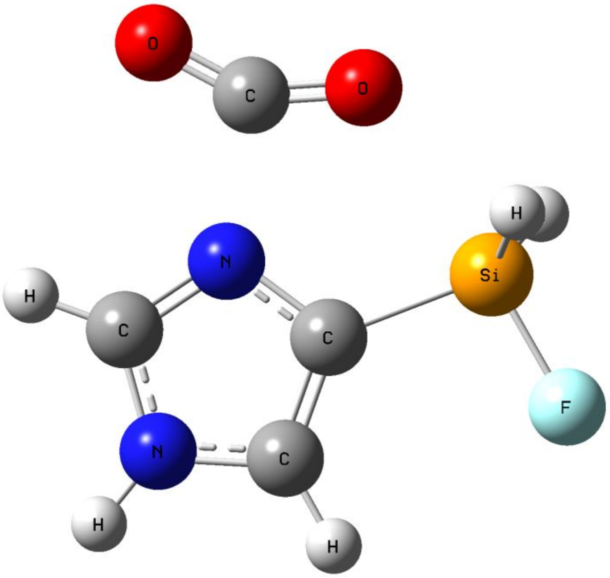	**Imi_4_SiH2F/CO2**<br>EMP2=-803.56684 NIMAG=1<br>C,2.01103151,0.19759337,-0.0<br>O,1.7287091573,-0.9832292126,-0.0<br>O,2.8587070445,1.0371822626,-0.0<br>C,0.0122061915,2.2482429645,-0.0<br>N,0.3878373221,0.9808223938,-0.00<br>C,-0.7172397989,0.1693064641,-0.00<br>C,-1.8195391407,1.0015456302,-0.00<br>N,-1.3408685132,2.2878452424,-0.00<br>H,-1.8980281245,3.1284085308,-0.00<br>H,-2.8723975693,0.7800314767,-0.00<br>H,0.664627015,3.1043733271,-0.00<br>Si,-0.5953090741,-1.7062958832,-0.00<br>F,-2.1943891469,-2.0637595065,-0.00<br>H,-0.08834348,-2.28854252,1.2474372<br>H,-0.08834348,-2.28854252,-1.2474372

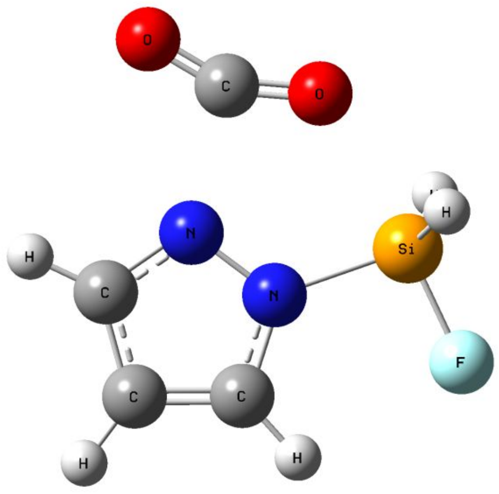	**Pz_1_SiH2F/CO2**<br>EMP2= -803.56928 NIMAG=1<br>C,2.106743212,0.0643916348,0.<br>O,1.6756946479,-1.0623364929,0.<br>O,2.9643302168,0.8709235008,0.<br>C,-0.0329785835,2.2858202779,0.<br>N,0.4020303517,1.0187218721,0.<br>N,-0.6706012463,0.2127435811,0.<br>C,-1.8097723902,0.9671105943,0.<br>C,-1.4328099852,2.298702133,0.<br>H,-2.0842359159,3.1550991338,0.0<br>H,-2.7767821606,0.4952595156,0.<br>H,0.6810391613,3.0928811416,0.<br>Si,-0.5654432307,-1.5992663698,0.<br>F,-2.1715865068,-1.8601365721,0.<br>H,-0.11848383,-2.16746597,-1.2702408<br>H,-0.11848383,-2.16746597,1.2702409
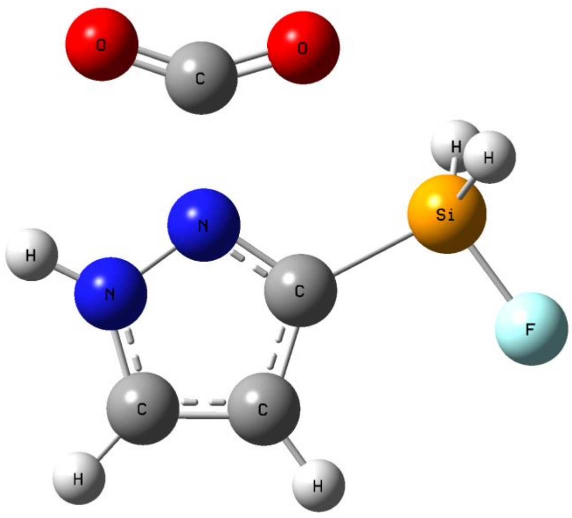	**Pz_3_SiH2F/CO2**<br>EMP2= -803.54402 NIMAG=1<br>C,1.9069935809,0.2848289965,0.<br>O,1.7729239606,-0.9277909528,0.<br>O,2.6993757243,1.1948139846,0.<br>N,0.0399513836,2.1717231978,0.<br>N,0.3498365261,0.8885023131,0.<br>C,-0.7655317711,0.1439169279,0.<br>C,-1.8485878227,1.043398965,0.<br>C,-1.3074362279,2.3224859933,0.<br>H,-1.7577427298,3.3002060944,0.<br>H,-2.8941993503,0.7887124836,0.<br>H,0.8007203682,2.8419622848,0.<br>Si,-0.6108202391,-1.7373100219,0.0<br>H,-0.072104,-2.28443447,1.24915204<br>H,-0.072104,-2.28443447,-1.2491520<br>F,-2.1926154098,-2.1415993012,0.

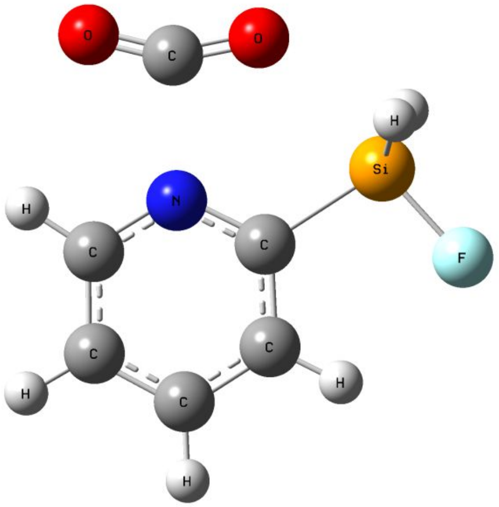	**Py_SiH2F/CO2**<br>EMP2= -825.55060 NIMAG=1<br>C,2.0253580458,-0.4391953816,0.<br>O,2.2910883066,-1.5926938787,0.<br>O,2.278106172,0.7333034347,0.<br>N,0.042922024,-0.368399738,0.<br>C,-0.6701674992,-1.4953442053,0.<br>C,-0.5399678181,0.8441241902,0.<br>C,-2.0603661989,-1.4726568474,0.<br>C,-1.9382952657,0.9259152831,0.<br>C,-2.7026412399,-0.2385413096,0.<br>H,-0.0920590204,-2.4122895478,0.<br>H,-2.6179808809,-2.3989877441,0.<br>H,-2.4104424035,1.898737536,0.<br>H,-3.7833267624,-0.1827980667,0.<br>Si,0.5140782934,2.4333568326,0.<br>F,-0.68330436,3.54810722,0.<br>H,1.24461546,2.68384205,1.2485088<br>H,1.24461546,2.68384205,-1.2485088
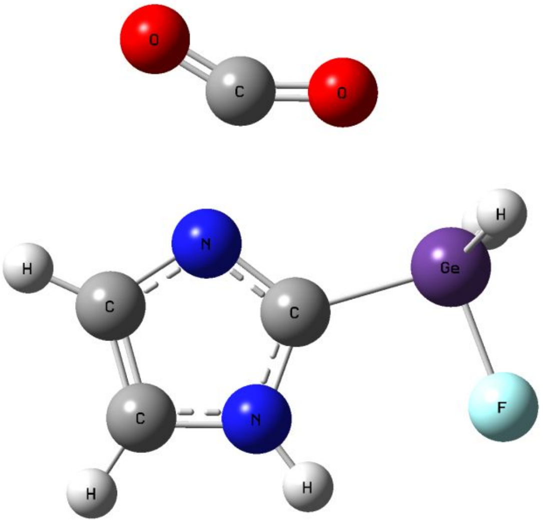	**Imi_2_GeH2F/CO2**<br>EMP2=-2590.09092 NIMAG=1<br>C,2.0769409,0.16740164,-0.017249758<br>O,1.75177167,-1.00152212,-0.020498<br>O,2.93248172,0.99014421,-0.01903083<br>N,0.42470863,1.02485985,-0.00209063<br>C,-0.63298393,0.2181985,0.001595693<br>N,-1.74621316,0.99043747,0.01422059<br>C,-1.38320109,2.30999780,0.01898263<br>C,-0.00432227,2.31895863,0.00849732<br>H,-2.10324341,3.10905967,0.02853471<br>H,-2.68194155,0.60897421,0.01987031<br>H,0.6814916,3.1486889,0.00783524<br>Ge,-0.58437097,-1.70496673,-0.009642<br>F,-2.3522063,-1.87620441,0.00256119<br>H,-0.17477955,-2.34152736,-1.3107074<br>H,-0.1554724,-2.35751819,1.27712062

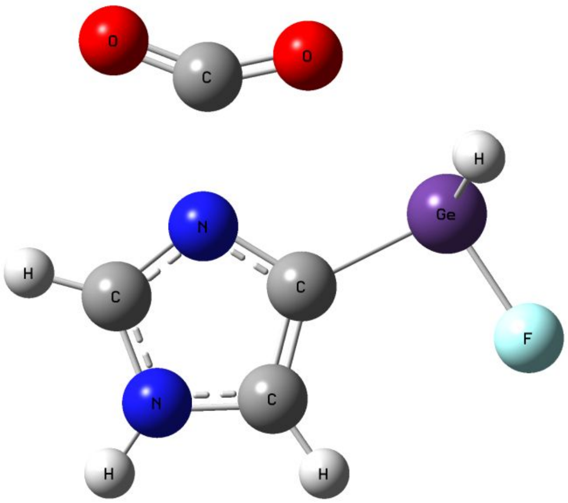	**Imi_4_GeH2F/CO2**<br>EMP2=-2590.08600 NIMAG=1<br>C,2.0122777,0.2461025,0.00641670<br>O,1.78406924,-0.9486077,0.00594396<br>O,2.84006452,1.10884542,0.00868013<br>C,0.02751357,2.24925124,0.00088396<br>N,0.39635496,0.97859636,0.00127739<br>C,-0.7117936,0.17656096,-0.00171673<br>C,-1.81026249,1.00872901,-0.0042081<br>N,-1.3243338,2.29307286,-0.0026466<br>H,-1.87700603,3.1366979,-0.00354789<br>H,-2.86278781,0.78645395,-0.006863<br>H,0.68616731,3.10044818,0.00252296<br>Ge,-0.58321781,-1.74589374,-0.001127<br>F,-2.3188020,-2.0742724,-0.0064300<br>H,-0.1007451,-2.35557651,-1.29158044<br>H,-0.10883876,-2.3554259,1.2923952
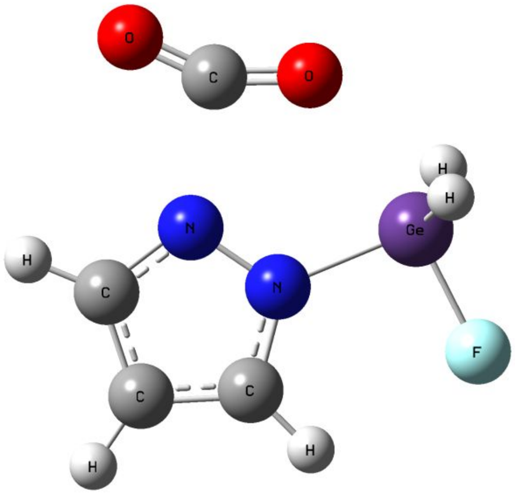	**Pz_1_GeH2F/CO2**<br>EMP2=-2590.08268 NIMAG=1<br>C,2.1236468,0.11634485,0.00383825<br>O,1.74778062,-1.03033489,0.00715688<br>O,2.9509129,0.95567254,0.00281071<br>C,-0.02863029,2.29066056,-0.0042170<br>N,0.40104343,1.01930212,-0.00306439<br>N,-0.67413453,0.22334535,-0.0007806<br>C,-1.8100613,0.97543448,-0.0006245<br>C,-1.42719323,2.30753764,-0.0029200<br>H,-2.0755371,3.16627825,-0.00348083<br>H,-2.77656069,0.50156620,0.00074976<br>H,0.68949645,3.09399713,-0.00605579<br>Ge,-0.55225804,-1.6621779,0.0016656<br>F,-2.28650807,-1.89927085,-0.0017881<br>H,-0.11304797,-2.22775980,-1.3184008<br>H,-0.1202891,-2.22561365,1.32511077

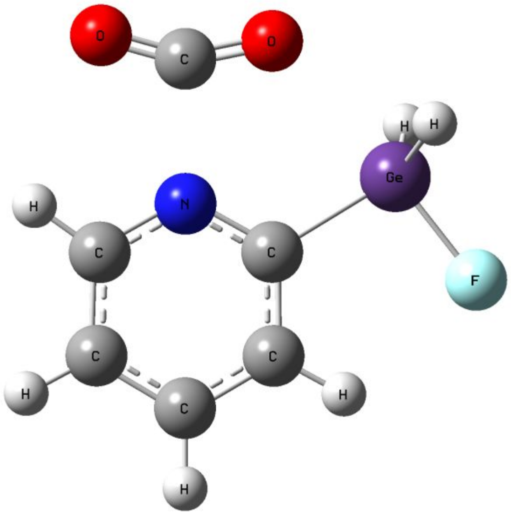	**Py_GeH2F/CO2**<br>EMP2=-2612.07138 NIMAG=1<br>C,1.9916312499,-0.475691989,0.<br>O,2.2482962519,-1.6334704703,0.<br>O,2.292589403,0.6886206592,0.<br>N,0.0460409708,-0.376308754,0.<br>C,-0.6650551819,-1.5056192167,0.<br>C,-0.539986648,0.8303135283,0.<br>C,-2.0544853443,-1.4804065429,0.<br>C,-1.9344846296,0.9190681016,0.<br>C,-2.6973293997,-0.2461418666,0.<br>H,-0.0855578846,-2.4211771988,0.<br>H,-2.6118970423,-2.4067293814,0.<br>H,-2.3979898837,1.8960058871,0.<br>H,-3.7779659762,-0.1912120392,0.<br>Ge,0.5557161862,2.4478146837,0.<br>F,-0.772279923,3.6116418003,0.<br>H,1.27249508,2.74680733,1.29226998<br>H,1.27249508,2.74680733,-1.29226998

Table S2. Relative energy (kJ mol^{-1}) of the stationary points in the reaction with respect to the isolated FLP + CO_2

FLP	FLP:CO_2	FLP/CO_2	FLP-CO_2
Imi_2_Si	-25.8	-2.7	-2.7
Imi_4_Si	-23.3	9.5	3.6
Pz_1_Si	-21.4	6.8	-24.2
Pz_3_Si	-22.3	23.3	23.1
Py_Si	-21.9	-2.9	-17.7
Imi_2_Ge	-30.3	-6.9	-15.4
Imi_4_Ge	-26.8	4.8	2.8
Pz_1_Ge	-25.5	-4.0	-27.8
Pz_3_Ge	-24.9	-	-
Py_Ge	-24.7	-8.1	-13.9

Fig. S1. Electronic energy vs. Free energy profile (kJ/mol). Full symbols represent the electronic energy and empty ones, the free energy.

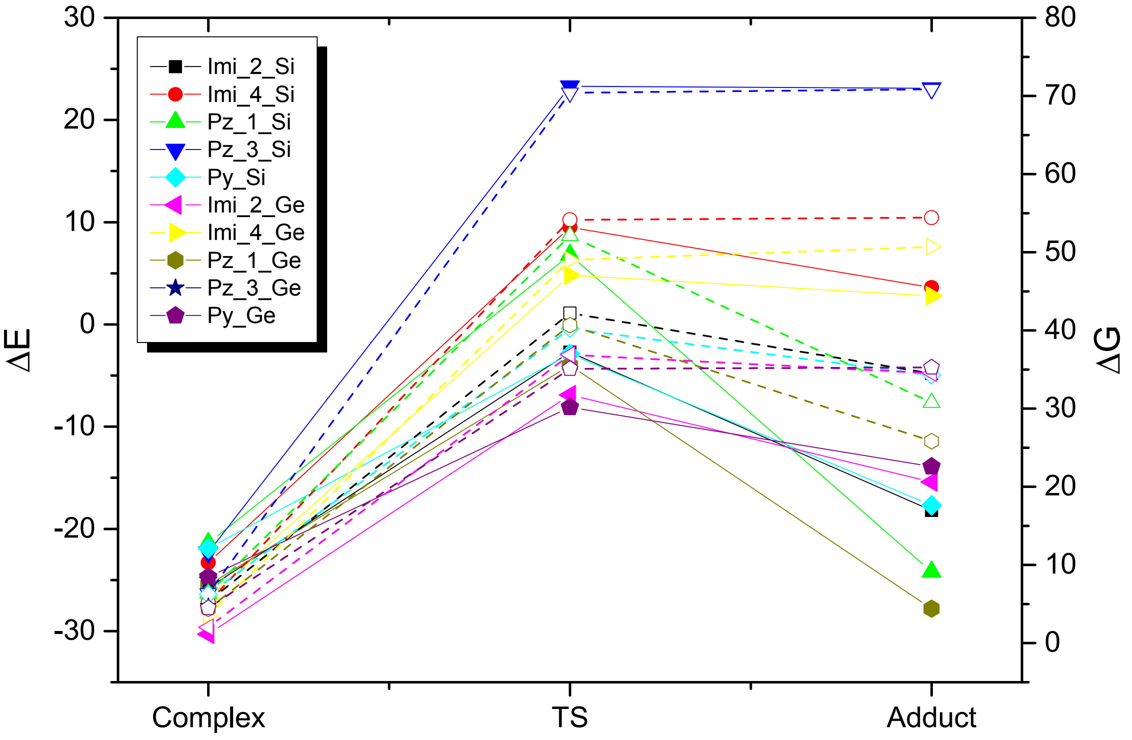

Fig. S2. Evolution of the N-C, O(1)-Si/Ge, C-O(1), and C-O(2) distances (in Å) along the reaction coordinate Imi_2_Ge (black); Imi_4_Ge (red); Pz_1_Ge (blue); Py_Ge (green).

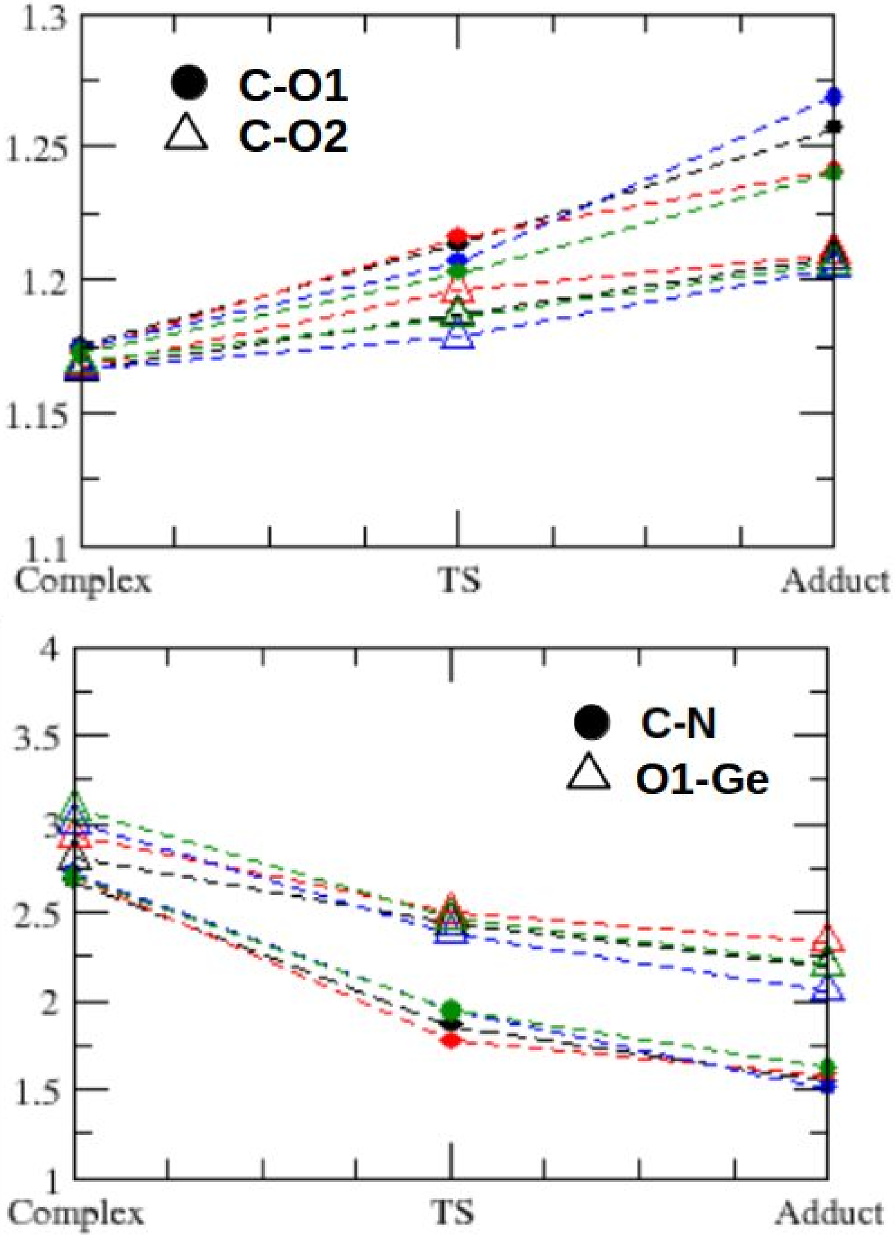

Table S3. γ and β parameters (see Eq. 1 and Eq. 2 of the main text).

FLP	γ	β
Imi_2_Si	0.20	0.32
Imi_4_Si	0.69	0.49
Pz_1_Si	-0.05	0.36
Pz_3_Si	0.99	0.81
Py_Si	0.13	0.33
Imi_2_Ge	0.46	0.39
Imi_4_Ge	0.88	0.61
Pz_1_Ge	-0.05	0.29
Pz_3_Ge	---	---
Py_Ge	0.48	0.40

Table S4. Electron density properties (au) at the intermolecular BCP's calculated in vacuo.

SYSTEM	BCP	RHO	LAP	G	V	H	|V|/G
**IMI_2_Si complex**	Si12-O2	0.010	0.034	0.008	-0.007	0.001	0.87
	N4-C1	0.017	0.057	0.013	-0.012	0.001	0.92
**IMI_2_Si TS**	N4-C1	0.098	0.057	0.054	-0.094	-0.040	1.74
**IMI_2_Si adduct**	O2-Si12	0.055	0.206	0.068	-0.084	-0.016	1.24
	N4-C1	0.229	-0.562	0.109	-0.359	-0.250	3.29
**IMI_4_Si complex**	N5-C1	0.016	0.054	0.012	-0.011	0.001	0.90
**IMI_4_Si TS**	N5-C1	0.120	0.005	0.063	-0.125	-0.062	1.98
**IMI_4_Si adduct**	O2-Si12	0.045	0.122	0.044	-0.058	-0.014	1.31
	N5-C1	0.216	-0.481	0.103	-0.327	-0.224	3.16
**PZ_1_Si complex**	N5-C1	0.014	0.049	0.011	-0.010	0.001	0.89
**PZ_1_Si TS**	Si12-O2	0.031	0.067	0.023	-0.030	-0.007	1.29
	N5-C1	0.085	0.087	0.050	-0.079	-0.029	1.57
**PZ_1_Si adduct**	O2-Si12	0.070	0.324	0.102	-0.122	-0.021	1.20
	N5-C1	0.242	-0.644	0.125	-0.410	-0.286	3.29
**PZ_3_Si complex**	N5-C1	0.015	0.054	0.012	-0.011	0.001	0.90
**PZ_3_Si TS**	Si12-O2	0.023	0.053	0.015	-0.017	-0.002	1.14
	N5-C1	0.160	-0.148	0.081	-0.200	-0.118	2.45
**PZ_3_Si adduct**	Si12-O2	0.029	0.055	0.019	-0.024	-0.005	1.28
	N5-C1	0.190	-0.318	0.095	-0.270	-0.175	2.83
**PY_Si complex**	N4-C1	0.015	0.050	0.011	-0.010	0.001	0.91
**PY_Si TS**	Si14-O3	0.024	0.059	0.018	-0.020	-0.003	1.16
	N4-C1	0.082	0.082	0.047	-0.073	-0.026	1.56
**PY_Si adduct**	Si14-O3	0.054	0.200	0.065	-0.081	-0.015	1.24
	N4-C1	0.207	-0.405	0.090	-0.282	-0.191	3.12
**IMI_2_Ge complex**	Ge12-O2	0.013	0.048	0.010	-0.009	0.001	0.86
	N4-C1	0.017	0.058	0.014	-0.013	0.001	0.92
**IMI_2_Ge TS**	Ge12-O2	0.030	0.098	0.026	-0.027	-0.001	1.05
	N4-C1	0.105	0.041	0.057	-0.103	-0.046	1.82
**IMI_2_Ge adduct**	O2-Ge12	0.054	0.165	0.053	-0.064	-0.012	1.22
	N4-C1	0.216	-0.478	0.101	-0.322	-0.221	3.18
**IMI_4_Ge complex**	Ge12-O2	0.010	0.038	0.008	-0.007	0.001	0.83
	N5-C1	0.016	0.056	0.013	-0.012	0.001	0.91
**IMI_4_Ge TS**	O2-Ge12	0.027	0.086	0.022	-0.023	-0.001	1.03
	N5-C1	0.128	-0.022	0.066	-0.137	-0.071	2.08
**IMI_4_Ge adduct**	O2-Ge12	0.040	0.121	0.035	-0.040	-0.005	1.14
	N5-C1	0.197	-0.364	0.093	-0.277	-0.184	2.97

**PZ_1_Ge complex**	Ge12-O2	0.009	0.034	0.007	-0.006	0.001	0.82
	N5-C1	0.016	0.055	0.013	-0.011	0.001	0.91
**PZ_1_Ge TS**	O2-Ge12	0.033	0.110	0.030	-0.032	-0.002	1.07
	N5-C1	0.087	0.083	0.051	-0.081	-0.030	1.59
**PZ_1_Ge adduct**	O2-Ge12	0.074	0.243	0.085	-0.109	-0.024	1.29
	N5-C1	0.236	-0.606	0.119	-0.389	-0.270	3.28
**PZ_3_Ge complex**	Ge12-O2	0.009	0.033	0.007	-0.006	0.001	0.81
	N5-C1	0.016	0.055	0.012	-0.011	0.001	0.90
**PY_Ge complex**	Ge14-O3	0.008	0.029	0.006	-0.005	0.001	0.80
	N4-C1	0.017	0.055	0.013	-0.012	0.001	0.93
**PY_Ge TS**	Ge14-O3	0.028	0.092	0.024	-0.024	-0.001	1.02
	N4-C1	0.089	0.070	0.049	-0.081	-0.032	1.64
**PY_Ge adduct**	Ge14-O3	0.052	0.164	0.051	-0.061	-0.010	1.20
	N4-C1	0.186	-0.283	0.082	-0.235	-0.153	2.86

Table S5. Classification of the tetrel bonds contacts based on the BCP properties

System	Complex	TS	Adduct
Imi_2_Si	N-C : CSI<br>Si-O : CSI	N-C : CSI-COV<br>Si-O : *No BCP*	N-C : COV<br>Si-O : CSI-COV
Imi_4_Si	N-C : CSI<br>Si-O : *no BCP*	N-C : CSI-COV<br>Si-O : *No BCP*	N-C : COV<br>Si-O : CSI-COV
Pz_1_Si	N-C : CSI<br>Si-O : *no BCP*	N-C : CSI-COV<br>Si-O : CSI-COV	N-C : COV<br>Si-O : CSI-COV
Pz_3_Si	N-C : CSI<br>Si-O : *no BCP*	N-C : COV<br>Si-O : CSI-COV	N-C : COV<br>Si-O : CSI-COV
Py_Si	N-C : CSI<br>Si-O : *no BCP*	N-C : CSI-COV<br>Si-O : CSI-COV	N-C : COV<br>Si-O : CSI-COV
Imi_2_Ge	N-C : CSI<br>Ge-O : CSI	N-C : CSI-COV<br>Ge-O : CSI-COV	N-C : COV<br>Ge-O : CSI-COV
Imi_4_Ge	N-C : CSI<br>Ge-O : CSI	N-C : COV<br>Ge-O : CSI-COV	N-C : COV<br>Ge-O : CSI-COV
Pz_1_Ge	N-C : CSI<br>Ge-O : CSI	N-C : CSI-COV<br>Ge-O : CSI-COV	N-C : COV<br>Ge-O : CSI-COV
Py_Ge	N-C : CSI<br>Ge-O : CSI	N-C : CSI-COV<br>Ge-O : CSI-COV	N-C : COV<br>Ge-O : CSI-COV

Fig. S3. ρ_{BCP} (au) vs. interactomic distance (Å) in the C-N, Si-O and Ge-O contacts along the reaction coordinate.

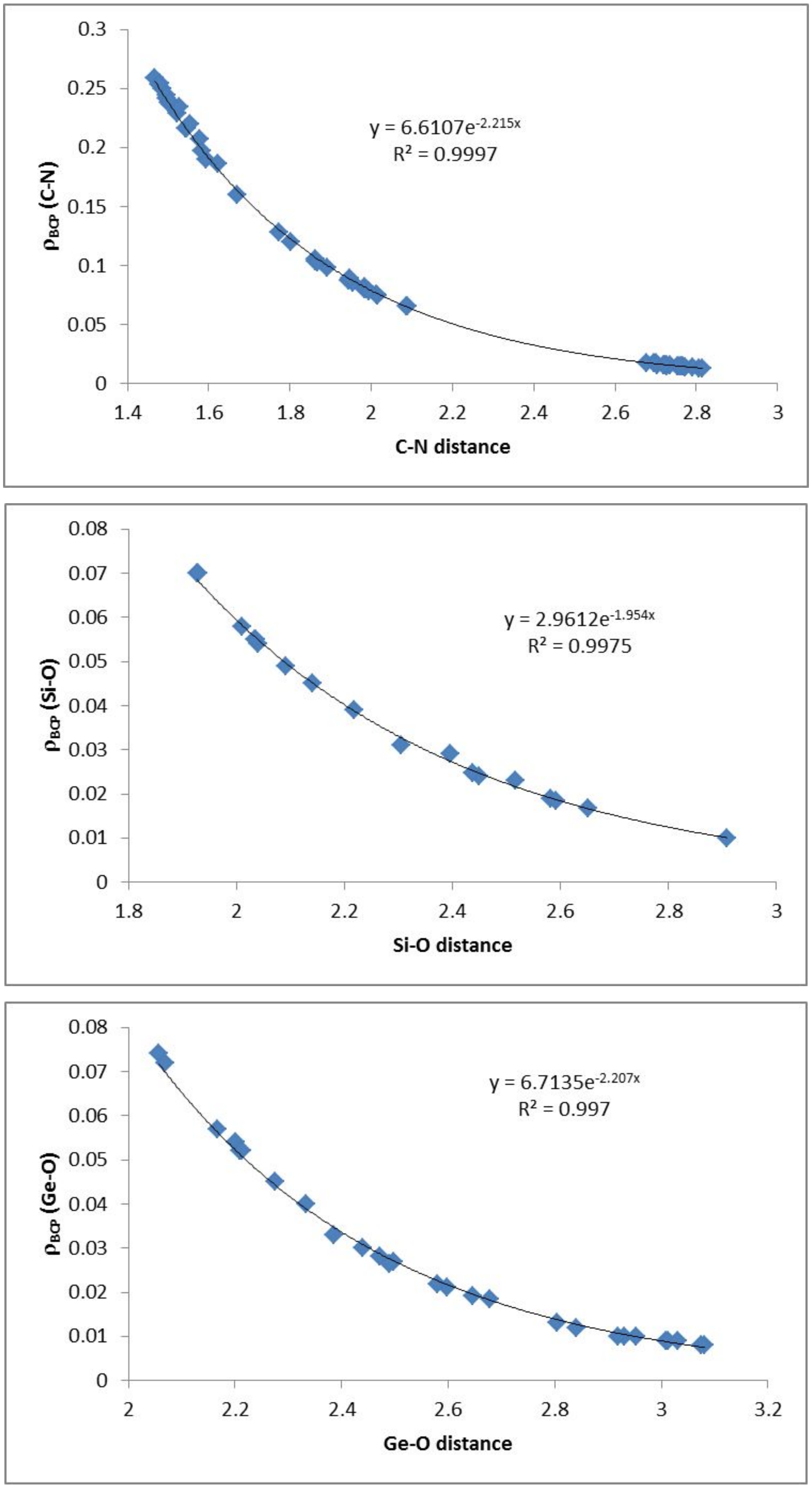

Table S6. NBO charge transfer stabilization energies (in kcal/mol).

System	***Orbital Interaction***	***Complex***	***TS***	***Adduct***
Imi_2_Si	LP(O)→BD*(Si-C)	0.68	5.52	12.37
	LP(O)→BD*(Si-H)	0.36	3.24	7.86
	LP(O)→BD*(Si-F)	3.43	14.65	81.87
	**TOTAL INTERACTION Si-O**	**4.83**	**26.65**	**109.96**
	LP(N)→BD*(C-O)	4.35	130.75	
Imi_4_Si	LP(O)→BD*(Si-C)	0.33	4.05	13.02
	LP(O)→BD*(Si-H)	0.17	2.57	8.22
	LP(O)→BD*(Si-F)	2.15	12.29	29.35
	**TOTAL INTERACTION Si-O**	**2.82**	**21.48**	**58.81**
	LP(N)→BD*(C-O)	3.94	166.28	
Pz_1_Si	LP(O)→BD*(Si-N)	0.12	7.83	32.36
	LP(O)→BD*(Si-H)	0.06	4.26	13.65
	LP(O)→BD*(Si-F)	1.28	15.17	40.51
	**TOTAL INTERACTION Si-O**	**1.52**	**31.52**	**100.17**
	LP(N)→BD*(C-O)	3.08	107.4	
Pz_3_Si	LP(O)→BD*(Si-N)	0.22	3.22	5.30
	LP(O)→BD*(Si-H)	0.07	2.03	3.42
	LP(O)→BD*(Si-F)	1.57	10.44	15.02
	**TOTAL INTERACTION Si-O**	**1.93**	**17.72**	**27.16**
	LP(N)→BD*(C-O)	3.42		
Py_Si	LP(O)→BD*(Si-C)	0.06	2.48	11.66
	LP(O)→BD*(Si-H)		1.76	7.80
	LP(O)→BD*(Si-F)	1.74	18.29	76.56
	**TOTAL INTERACTION Si-O**	**1.8**	**24.29**	**103.82**
	LP(N)→BD*(C-O)	3.80	157.49	
Imi_2_Ge	LP(O)→BD*(Ge-C)	1.15	5.36	15.21
	LP(O)→BD*(Ge-H)	0.58	2.64	7.03
	LP(O)→BD*(Ge-F)	5.12	15.01	33.15
	**TOTAL INTERACTION Ge-O**	**7.43**	**25.65**	**62.42**
	LP(N)→BD*(C-O)	4.57	145.76	
Imi_4_Ge	LP(O)→BD*(Ge-C)	0.66	4.11	8.56
	LP(O)→BD*(Ge-H)	0.35	2.25	4.62
	LP(O)→BD*(Ge-F)	3.63	12.98	22.76

	**TOTAL INTERACTION Ge-O**	**4.99**	**21.59**	**40.56**
	LP(N)→BD*(C-O)	4.21		
Pz_1_Ge	LP(O)→BD*(Ge-N)	0.53	7.74	31.89
	LP(O)→BD*(Ge-H)	0.27	3.53	10.89
	LP(O)→BD*(Ge-F)	2.80	15.24	39.01
	**TOTAL INTERACTION Ge-O**	**3.87**	**30.04**	**92.68**
	LP(N)→BD*(C-O)	3.77	111.05	
Py_Ge	LP(O)→BD*(Ge-C)	0.17	2.84	8,66
	LP(O)→BD*(Ge-H)	0.09	1.76	4.92
	LP(O)→BD*(Ge-F)	3.54	21.10	55.96
	**TOTAL INTERACTION Ge-O**	**3.89**	**27.46**	**74.36**
	LP(N)→BD*(C-O)	10.01	180.44	

Table S7. Relative energy (kJ mol^{-1}) of the stationary points in the reaction with respect to the isolated FLP + CO_2 including the effect of the solvent [PCM(Acetonitrile)].

FLP	FLP:CO_2	FLP/CO_2	FLP-CO_2
Imi_2_Si	-15.8	0.5	-41.8
Imi_4_Si	-15.3	3.6	-30.5
Pz_1_Si	-14.2	12.5	-36.8
Pz_3_Si	-11.1	20.2	4.9
Py_Si	-15.3	-1.4	-41.0

FLP	FLP:CO_2	FLP/CO_2	FLP-CO_2
Imi_2_Ge	-19.5	-4.0	-38.3
Imi_4_Ge	-18.5	-0.7	-29.0
Pz_1_Ge	-10.9	9.3	-31.8
Pz_3_Ge	-14.4	16.7	3.6
Py_Ge	-17.9	-7.0	-35.1

Table S8. γ and β parameters for the reactions in PCM(acetonitrile).

FLP	γ	β
Imi_2_Si	-0.45	0.05
Imi_4_Si	-0.28	0.09
Pz_1_Si	-0.30	0.24
Pz_3_Si	0.002	0.25
Py_Si	-0.48	0.10
Imi_2_Ge	-0.38	0.08
Imi_4_Ge	-0.23	0.13
Pz_1_Ge	-0.34	0.17
Pz_3_Ge	0.41	0.64
Py_Ge	-0.44	0.09

Table S9. Electron density properties (au) at the intermolecular BCP's calculated using the PCM(acetonitrile) model.

SYSTEM	BCP	RHO	LAP	G	V	H	|V|/G
**IMI_2_Si complex**	N4-C1	0.015	0.051	0.012	-0.011	0.001	0.90
**IMI_2_Si TS**	Si12-O2	0.019	0.053	0.014	-0.014	0.000	1.03
	N4-C1	0.075	0.092	0.044	-0.065	-0.021	1.48
**IMI_2_Si adduct**	O2-Si12	0.055	0.206	0.068	-0.084	-0.016	1.24
	N4-C1	0.254	-0.724	0.124	-0.430	-0.305	3.46
**IMI_4_Si complex**	N5-C1	0.015	0.050	0.011	-0.010	0.001	0.90
**IMI_4_Si TS**	Si12-O2	0.017	0.048	0.012	-0.012	0.000	1.01
	N5-C1	0.080	0.087	0.047	-0.072	-0.025	1.54
**IMI_4_Si adduct**	O2-Si12	0.049	0.159	0.055	-0.070	-0.015	1.27
	N5-C1	0.250	-0.698	0.123	-0.420	-0.297	3.42
**PZ_1_Si complex**	N5-C1	0.013	0.046	0.010	-0.009	0.001	0.88
**PZ_1_Si TS**	Si12-O2	0.025	0.059	0.018	-0.020	-0.003	1.16
	N5-C1	0.080	0.092	0.048	-0.072	-0.025	1.52
**PZ_1_Si adduct**	O2-Si12	0.070	0.322	0.101	-0.122	-0.021	1.20
	N5-C1	0.259	-0.748	0.138	-0.462	-0.325	3.36
**PZ_3_Si complex**	N5-C1	0.014	0.050	0.011	-0.010	0.001	0.88
**PZ_3_Si TS**	N5-C1	0.104	0.050	0.058	-0.104	-0.045	1.78
**PZ_3_Si adduct**	Si12-O2	0.039	0.080	0.032	-0.045	-0.012	1.38
	N5-C1	0.238	-0.619	0.124	-0.402	-0.278	3.25
**PY_Si complex**	N4-C1	0.014	0.047	0.011	-0.010	0.001	0.90
**PY_Si TS**	Si14-O3	0.019	0.053	0.014	-0.014	-0.001	1.04
	N4-C1	0.065	0.096	0.039	-0.054	-0.015	1.39
**PY_Si adduct**	Si14-O3	0.058	0.231	0.074	-0.091	-0.016	1.22
	N4-C1	0.234	-0.571	0.099	-0.341	-0.242	3.44
**IMI_2_Ge complex**	Ge12-O2	0.012	0.045	0.010	-0.008	0.001	0.85
	N4-C1	0.016	0.054	0.012	-0.011	0.001	0.91
**IMI_2_Ge TS**	Ge12-O2	0.021	0.071	0.017	-0.016	0.001	0.96
	N4-C1	0.075	0.091	0.044	-0.066	-0.022	1.49
**IMI_2_Ge adduct**	N4-C1	0.244	-0.667	0.117	-0.401	-0.284	3.42
	O2-Ge12	0.052	0.161	0.051	-0.062	-0.011	1.21
**IMI_4_Ge complex**	Ge12-O2	0.010	0.039	0.008	-0.007	0.001	0.83
	N5-C1	0.016	0.053	0.012	-0.011	0.001	0.90
**IMI_4_Ge TS**	O2-Ge12	0.019	0.065	0.015	-0.014	0.001	0.94
	N5-C1	0.080	0.087	0.046	-0.071	-0.025	1.53
**IMI_4_Ge**	O2-Ge12	0.045	0.138	0.042	-0.049	-0.007	1.18

**adduct**	N5-C1	0.239	-0.635	0.115	-0.389	-0.274	3.38
**PZ_1_Ge complex**	Ge12-O2	0.009	0.032	0.007	-0.006	0.001	0.81
	N5-C1	0.015	0.050	0.011	-0.010	0.001	0.89
**PZ_1_Ge TS**	Ge12-O2	0.027	0.089	0.022	-0.023	0.000	1.01
	N5-C1	0.078	0.093	0.047	-0.070	-0.023	1.50
**PZ_1_Ge adduct**	O2-Ge12	0.072	0.235	0.082	-0.105	-0.023	1.28
	N5-C1	0.253	-0.712	0.131	-0.439	-0.309	3.36
**PZ_3_Ge complex**	C1-N5	0.013	0.047	0.010	-0.009	0.001	0.87
**PZ_3_Ge TS**	O2-Ge12	0.019	0.061	0.014	-0.014	0.001	0.94
	N5-C1	0.103	0.052	0.058	-0.102	-0.044	1.77
**PZ_3_Ge adduct**	O2-Ge12	0.010	0.037	0.008	-0.006	0.001	0.82
	N5-C1	0.015	0.052	0.012	-0.010	0.001	0.89
**PY_Ge complex**	Ge14-O3	0.008	0.030	0.006	-0.005	0.001	0.80
	N4-C1	0.016	0.053	0.012	-0.011	0.001	0.92
**PY_Ge TS**	Ge14-O3	0.022	0.075	0.018	-0.017	0.001	0.96
	N4-C1	0.066	0.095	0.039	-0.055	-0.016	1.40
**PY_Ge adduct**	Ge14-O3	0.057	0.184	0.059	-0.072	-0.013	1.22
	N4-C1	0.220	-0.485	0.093	-0.307	-0.214	3.30

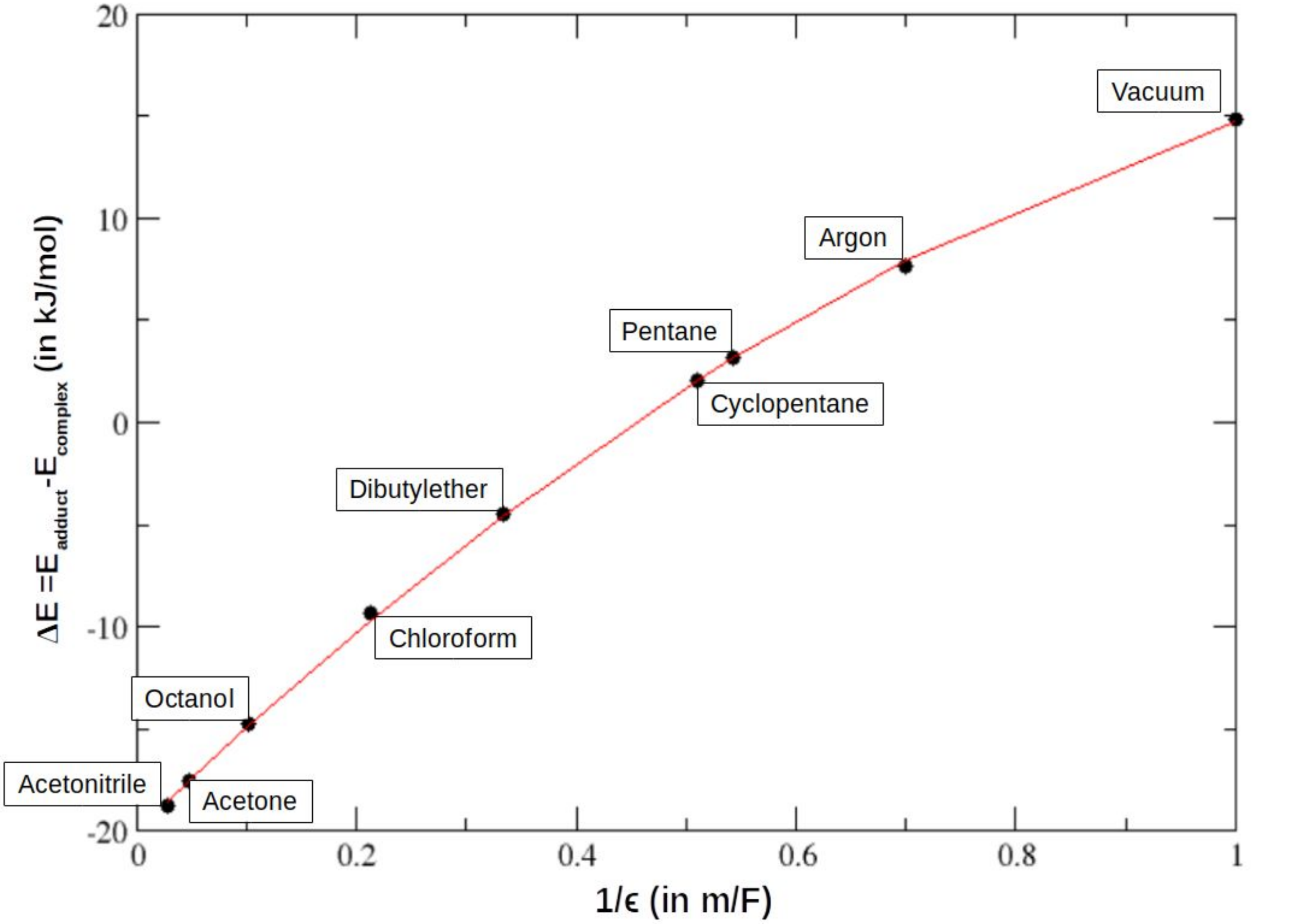

Fig. S4 Adduct-Complex energy difference (kJ mol^{-1}) for the Imi_2_Ge + CO_2 reaction as a function of the inverse of the dielectric constant of the solvent. The fitted equation is $\Delta E = -17.25\ (1/\epsilon)^2 + 51.97\ (1/\epsilon) - 19.99$, $R^2 = 1.00$.

Table S10: Geometries and energies of the OH-substituted molecules.

**Pz_1_GeH2F_OH**	
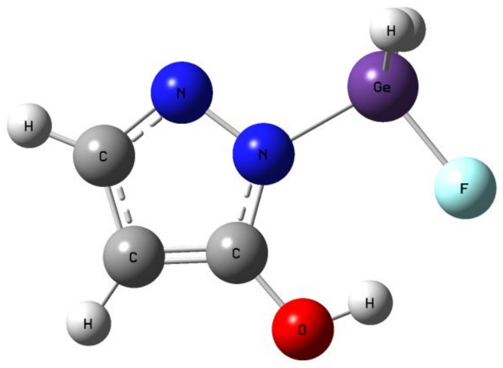	**Pz_1_GeH2F_OH**<br>EMP2= -2476.88746 NIMAG=0<br>C,-0.10749485,2.20711993,-0.00156695<br>N,0.262988652,0.9202879641,0.00420753<br>N,-0.9182576063,0.22931522,0.00465154<br>C,-1.9932740372,1.06920482,-0.00185404<br>C,-1.5057105843,2.35917260,-0.00591672<br>H,-2.0869552223,3.2636362254,-0.012121<br>O,-3.2770529998,0.65109344,-0.00004077<br>H,0.6522106838,2.971707441,-0.00329593<br>Ge,-0.7095176673,-1.59375989,0.0146779<br>F,-2.385271355,-2.082542669,-0.02560229<br>H,-0.1231563758,-2.06796278,1.31842725<br>H,-0.0560952461,-2.06991457,-1.2556679<br>H,-3.3000333933,-0.31770773,-0.0210288
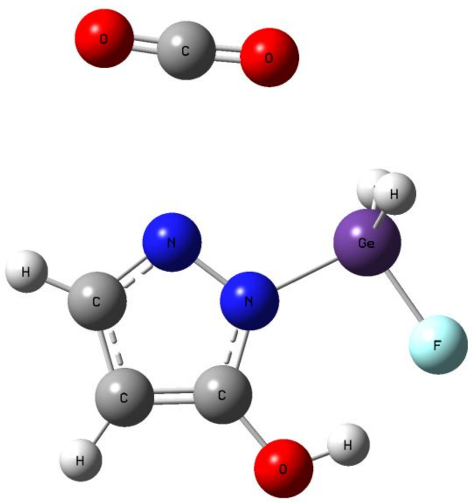	**Pz_1_GeH2F_OH:CO2**<br>EMP2= -2665.21951 NIMAG=0<br>C,2.6469605988,-0.81254834,0.43102084<br>N,1.3890208234,-1.2451433167,0.5812749<br>N,0.6094358052,-0.1717525395,0.243786<br>C,1.3777040246,0.9032582597,-0.1082441<br>C,2.6992024175,0.5230729948,0.0018371<br>H,3.5589199504,1.135479816,-0.20256997<br>O,0.8842641564,2.0986344232,-0.4873799<br>H,3.4604106016,-1.488451564,0.64062011<br>Ge,-1.2184134651,-0.41715484,0.3390465<br>F,-1.7225294509,1.181826808,-0.1696431<br>H,-1.6840009087,-0.583589314,1.7590001<br>H,-1.7062232682,-1.37630319,-0.7110949<br>H,-0.0854462465,2.06801561,-0.46898389<br>C,0.3622924574,-3.61899654,1.35266942<br>O,1.4051052643,-4.1178633555,1.5033782<br>O,-0.7191988602,-3.17902599,1.22120681

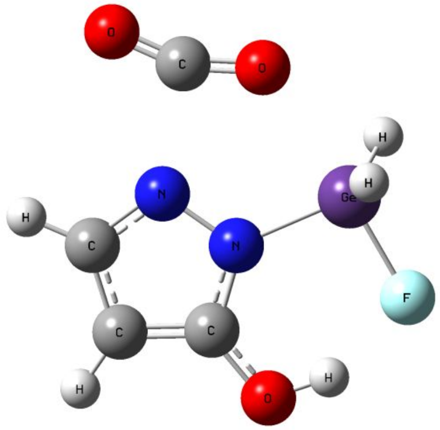	**Pz_1_GeH2F_OH/CO2**<br>EMP2= -2665.21409 NIMAG=1<br>C,2.6805375757,-0.90312071,0.45936163<br>N,1.4308605042,-1.3504651,0.61032814<br>N,0.599066051,-0.324616698,0.29311807<br>C,1.3439821868,0.771676520,-0.05870495<br>C,2.6812732192,0.429958326,0.03859764<br>H,3.5173533443,1.07261923,-0.17103494<br>O,0.8386069334,1.955963535,-0.42856377<br>H,3.5050269336,-1.567301925,0.66242558<br>Ge,-1.2624931947,-0.5602586,0.38257854<br>F,-1.6388913725,1.09391024,-0.14089845<br>H,-1.8114695336,-0.576024350,1.7797826<br>H,-1.8304388197,-1.378703470,-0.740513<br>H,-0.1347589672,1.907322764,-0.4072781<br>C,0.5306015941,-3.1024788949,1.1761351<br>O,1.4137134833,-3.8392254085,1.4016565<br>O,-0.605466038,-2.7297964986,1.0689336
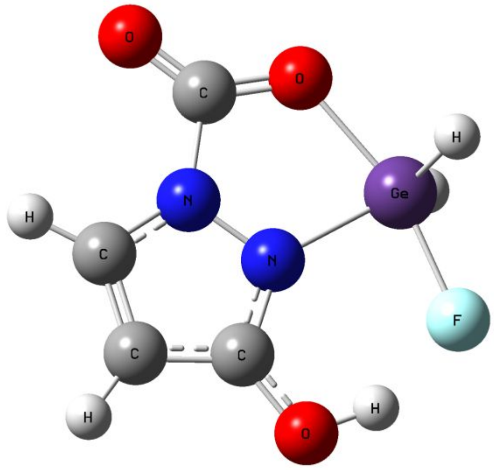	**Pz_1_GeH2F_OH-CO2**<br>EMP2= -2665.23294 NIMAG=0<br>C,2.5820354191,-1.249939766,0.66433173<br>N,1.3083206991,-1.657459291,0.80038161<br>N,0.4595007958,-0.635187256,0.59409831<br>C,1.2033467417,0.457685636,0.31838404<br>C,2.5597184843,0.0992549171,0.355360<br>H,3.3964155594,0.7506210603,0.1781071<br>O,0.6855017609,1.6463160533,0.0630981<br>H,3.3866680116,-1.95266234,0.79898485<br>Ge,-1.418797576,-1.07897781,0.75708692<br>F,-1.6766471631,0.699671533,0.35710883<br>H,-2.0097919605,-1.01472051,2.13855736<br>H,-2.0983505943,-1.6137921,-0.4733567<br>H,-0.2979334145,1.548438862,0.11781853<br>C,0.7006778073,-2.96174835,1.11792482<br>O,1.4337829563,-3.8981144647,1.3079157<br>O,-0.5764825571,-2.83669251,1.13091845

**Imi_4_GeH2F_OH**	
	**Imi_4_GeH2F_OH**<br>EMP2= -2476.89924 NIMAG=0<br>C,2.4645393957,-0.315656481,0.90643749<br>N,1.2095950796,-0.423218605,1.29291028<br>C,0.4528816355,-0.369803078,0.12918292<br>C,1.2959373903,-0.227758508,-0.9552414<br>N,2.5632144374,-0.19438756,-0.45579589<br>H,3.4020229444,-0.09828130,-1.00576456<br>O,1.1057502358,-0.126743551,-2.2840592<br>H,3.3295505337,-0.318288848,1.54771269

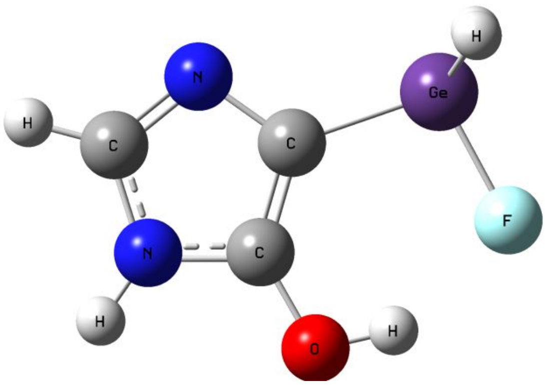	Ge,-1.4329045286,-0.47378445,0.034762<br>F,-1.6404211383,-0.33869994,-1.7169652<br>H,-2.1876589811,0.6956557449,0.6314117<br>H,-2.0396529098,-1.80641131,0.42093297<br>H,0.1454146055,-0.17163954,-2.43528737
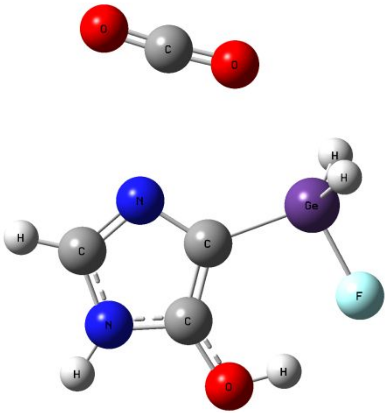	**Imi_4_GeH2F_OH:CO2**<br>EMP2= -2665.23278 NIMAG=0<br>C,-2.7056316658,-0.94965927,0.07849053<br>N,-1.4700983146,-1.40557227,0.13497031<br>C,-0.6427672886,-0.294531999,0.0305253<br>C,-1.4308954749,0.83572457,-0.08986665<br>N,-2.7261725733,0.411933737,-0.0585336<br>H,-3.5337686884,1.01100268,-0.12576233<br>O,-1.1735217205,2.14802908,-0.21944177<br>H,-3.6039184083,-1.54131156,0.13021128<br>Ge,1.2570483581,-0.272812156,0.0439227<br>F,1.4745938851,1.4848381,-0.13166488<br>H,1.9508090914,-0.858156881,-1.1644551<br>H,1.9305422021,-0.60359763,1.35582832<br>H,-0.2024950392,2.24114104,-0.22102764<br>C,-0.0925422122,-3.68236495,0.37564977<br>O,0.8780821303,-3.0229171338,0.3181131<br>O,-1.001230141,-4.410414576,0.44062729
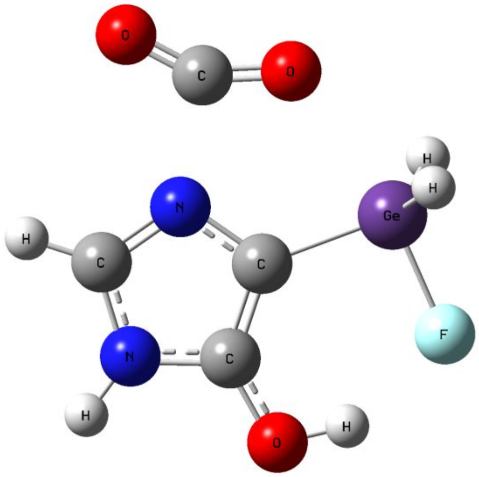	**Imi_4_GeH2F_OH/CO2**<br>EMP2= -2665.22364 NIMAG=1<br>C,-2.5748159837,-1.151013,0.39016246<br>N,-1.2954605323,-1.4687352,0.30211834<br>C,-0.5678395226,-0.34751492,0.0022062<br>C,-1.4607719782,0.69882748,-0.09469111<br>N,-2.7051053547,0.18475013,0.14897792<br>H,-3.5634860233,0.715196931,0.14762242<br>O,-1.3104900793,1.99868954,-0.36170164<br>H,-3.3868007104,-1.82063275,0.61318605<br>Ge,1.3223333455,-0.32793437,-0.2235743<br>F,1.3562871389,1.44215238,-0.56586865<br>H,1.8750150923,-0.91711098,-1.4953372<br>H,2.17069824,-0.4373326494,1.01667003<br>H,-0.3495047161,2.13218712,-0.49918015<br>C,-0.3299383399,-3.03430183,0.48848579<br>O,-1.0757349484,-3.923056038,0.7461277<br>O,0.8036485124,-2.652841091,0.28238277

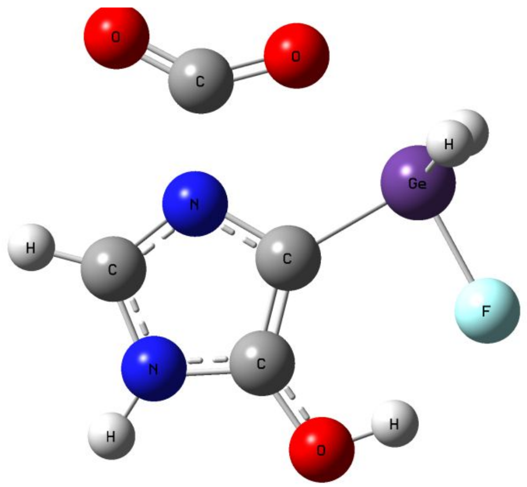

Imi_4_GeH2F_OH-CO2
EMP2= -2665.22615   NIMAG=0
C,-2.0273994278,1.45136307,0.00032877
N,-1.388995958,0.2890346002,0.00004297
C,0.1663028988,1.8254481803,0.0002678
O,1.2830997425,2.5457223632,0.0003832
H,-3.0921655132,1.598264347,0.00044142
Ge,1.116520045,-1.04910821,-0.00034811
F,2.5446803126,0.1088374116,-0.0002141
H,1.3595380552,-1.76262024,-1.30341351
H,1.3595243826,-1.7630524308,1.3024869
H,2.010584507,1.8819227831,0.00028309
C,-1.9155588963,-1.17837757,-0.0002924
O,-3.1188662146,-1.28693364,-0.0002165
O,-0.9197028437,-1.94057530,-0.0006289
C,-0.0384514259,0.46713770,0.00001653
N,-1.0710927111,2.4182921887,0.0003339
H,-1.241044793,3.4137633922,0.00040556

Py_GeH2F_OH

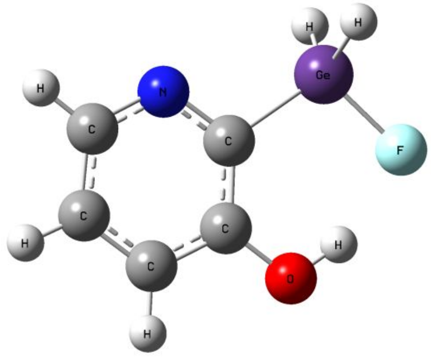

Py_GeH2F_OH
EMP2= -2498.88494   NIMAG=0
N,-1.06671494,-1.19626023,-0.0015128
C,-2.40182567,-1.10811545,-0.0019238
C,-0.35269281,-0.05046056,0.0005829
C,-3.07388225,0.11370729,-0.0003262
C,-0.94931162,1.21786683,0.00202867
C,-2.34308801,1.29313428,0.00162051
H,-2.94484125,-2.04482121,-0.0035661
H,-4.1554706,0.13793871,-0.00081672
H,-2.82093196,2.26417718,0.0026924
Ge,1.53274435,-0.4129750,0.00211827
F,2.2635890072,1.19680675,-0.002874
H,2.0145712071,-1.08371363,1.268758
H,2.01426896,-1.0925523,-1.25981117
O,-0.2525022408,2.37789637,0.004101
H,0.7019095046,2.18731,0.00155220

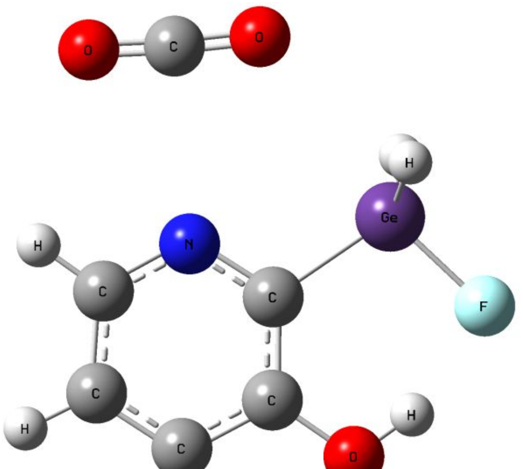

Py_GeH2F_OH:CO2
EMP2= -2687.21633   NIMAG=0
C,-1.4539710612,-2.5922847085,0.
O,-2.6182340133,-2.4935654154,0.
O,-0.2939901413,-2.7673438847,0.
N,-1.1323963141,0.0920751432,0.
C,-2.2783884265,0.7820586563,0.
C,0.0370653564,0.7689570319,0.

	C,-2.313030239,2.1753786533,0.<br>C,0.0849925873,2.173097324,0.<br>C,-1.1194689484,2.8806165366,0.<br>H,-3.1882616658,0.1942954971,0.<br>H,-3.2611373817,2.6962231469,0.<br>H,-1.0921318546,3.9625523772,0.<br>Ge,1.583938209,-0.3867636915,0.<br>F,2.9229304244,0.7749418455,0.<br>H,1.7592519,-1.18275624,1.27113057<br>H,1.7592519,-1.18275624,-1.27113057<br>O,1.2277717209,2.8939275424,0.<br>H,1.9963374629,2.2947274968,0.
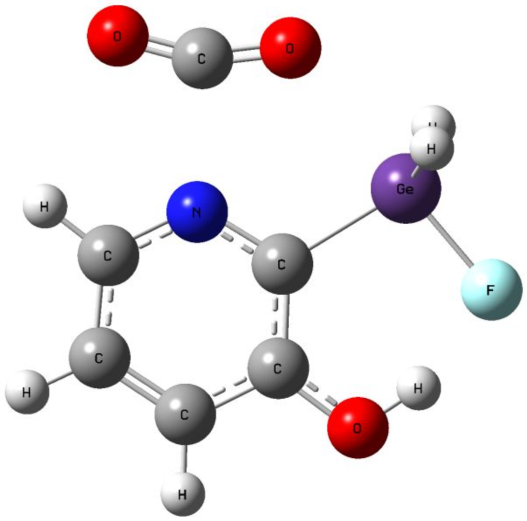	**Py_GeH2F_OH/CO2**<br>EMP2= -2687.21211 NIMAG=1<br>C,-1.3263786245,-2.0437084364,-0.<br>O,-2.4859443663,-2.2474497506,-0.<br>O,-0.1562733516,-2.2871953645,-0.<br>N,-1.216509934,0.036123547,-0.<br>C,-2.3568809519,0.7263440114,-0.<br>C,-0.0131440552,0.6395483592,-0.<br>C,-2.3551781639,2.1178486238,-0.<br>C,0.0540670881,2.0494187782,-0.<br>C,-1.1400021068,2.7812935304,-0.<br>H,-3.2651601599,0.1365208799,-0.<br>H,-3.2873702579,2.6659317068,-0.<br>H,-1.0787451827,3.8617502397,-0.<br>Ge,1.5563618438,-0.5265154628,-0.<br>F,2.8256331496,0.7403589059,-0.<br>H,1.85569949,-1.24044848,1.29283319<br>H,1.85569949,-1.24044848,-1.2928332<br>O,1.1932111809,2.7610550917,-0.<br>H,1.9614443843,2.1529533708,-0.
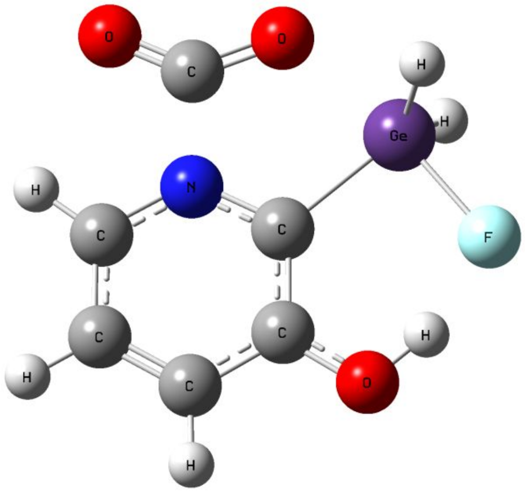	**Py_GeH2F_OH-CO2**<br>EMP2= -2687.22101 NIMAG=0<br>C,-1.2987462325,-1.5322437534,0.<br>O,-2.4179775344,-1.9778038016,0.<br>O,-0.1398660696,-2.0149142573,0.<br>N,-1.2095538804,0.0412728604,0.<br>C,-2.3567265341,0.7324867046,0.<br>C,0.0249065408,0.5807573677,0.<br>C,-2.2955509206,2.1182568053,0.<br>C,0.1159687688,1.9889562653,0.<br>C,-1.0632516603,2.7500623343,0.<br>H,-3.257426226,0.1366649963,0.

	H,-3.2124774396,2.6898478748,0.<br>H,-0.979092599,3.828841829,0.<br>Ge,1.4833050283,-0.7395438157,0.<br>F,2.7554558503,0.6114584384,0.<br>H,1.89824836,-1.3347542,1.32022454<br>H,1.89824836,-1.3347542,-1.32022454<br>O,1.2732045525,2.6376389918,0.<br>H,2.0095911749,1.9668090873,0.

Table S11. Relative energies (kJ mol^{-1}) of the stationary points with the inclusion of a hydroxyl group.

**FLP**	***Complex***	***TS***	***Adduct***
**Imi_4_OH**	-31.2	-7.3	-13.8
**Pz_1_OH**	-27.3	-13.1	-62.6
**Py_OH**	-25.6	-14.5	-37.9

Table S12. Selected geometrical parameters (Å) of the stationary points with the inclusion of a hydroxyl group.

	**Complex**		**TS**		**Adduct**	
	**C-N**	**O-Ge/Si**	**C-N**	**O-Ge/Si**	**C-N**	**O-Ge/Si**
**Imi_4_Ge_OH**	2.672	2.790	1.849	2.435	1.559	2.223
**Pz_1_Ge_OH**	2.699	2.942	2.049	2.368	1.474	1.985
**Py_Ge_OH**	2.704	3.032	2.083	2.456	1.576	2.064
